# The Impact of Stay-At-Home Mandates on Uncertainty and Sentiments: Quasi-Experimental Study

**DOI:** 10.2196/64667

**Published:** 2025-03-04

**Authors:** Carolina Biliotti, Nicolò Fraccaroli, Michelangelo Puliga, Falco J Bargagli-Stoffi, Massimo Riccaboni

**Affiliations:** 1 AXES IMT School for Advanced Studies Lucca Lucca Italy; 2 W R Rhodes Center for International Economics and Finance Brown University Providence, RI United States; 3 Bflows Network Lab Cagliari Italy; 4 Department of Biostatistics University of California, Los Angeles Los Angeles, CA United States; 5 Department of Biostatistics Harvard University Cambridge, MA United States; 6 Istituto Universitario di Studi Superiori di Pavia Pavia Italy

**Keywords:** lockdown policy, sentiment analysis, uncertainty, social media, quasi-experiment

## Abstract

**Background:**

As the spread of the SARS-CoV-2 virus coincided with lockdown measures, it is challenging to distinguish public reactions to lockdowns from responses to COVID-19 itself. Beyond the direct impact on health, lockdowns may have worsened public sentiment toward politics and the economy or even heightened dissatisfaction with health care, imposing a significant cost on both the public and policy makers.

**Objective:**

This study aims to analyze the causal effect of COVID-19 lockdown policies on various dimensions of sentiment and uncertainty, using the Italian lockdown of February 2020 as a quasi-experiment. At the time of implementation, communities inside and just outside the lockdown area were equally exposed to COVID-19, enabling a quasi-random distribution of the lockdown. Additionally, both areas had similar socioeconomic and demographic characteristics before the lockdown, suggesting that the delineation of the strict lockdown zone approximates a randomized experiment. This approach allows us to isolate the causal effects of the lockdown on public emotions, distinguishing the impact of the policy itself from changes driven by the virus’s spread.

**Methods:**

We used Twitter data (N=24,261), natural language models, and a difference-in-differences approach to compare changes in sentiment and uncertainty inside (n=1567) and outside (n=22,694) the lockdown areas before and after the lockdown began. By fine-tuning the AlBERTo (Italian BERT optimized) pretrained model, we analyzed emotions expressed in tweets from 1124 unique users. Additionally, we applied dictionary-based methods to categorize tweets into 4 dimensions—economy, health, politics, and lockdown policy—to assess the corresponding emotional reactions. This approach enabled us to measure the direct impact of local policies on public sentiment using geo-referenced social media and can be easily adapted for other policy impact analyses.

**Results:**

Our analysis shows that the lockdown had no significant effect on economic uncertainty (b=0.005, SE 0.007, t125=0.70; *P*=.48) or negative economic sentiment (b=–0.011, SE 0.0089, t125=–1.32; *P*=.19). However, it increased uncertainty about health (b=0.036, SE 0.0065, t125=5.55; *P*<.001) and lockdown policy (b=0.026, SE 0.006, t125=4.47; *P*<.001), as well as negative sentiment toward politics (b=0.025, SE 0.011, t125=2.33; *P*=.02), indicating that lockdowns have broad externalities beyond health. Our key findings are confirmed through a series of robustness checks.

**Conclusions:**

Our findings reveal that lockdowns have broad externalities extending beyond health. By heightening health concerns and negative political sentiment, policy makers have struggled to secure explicit public support for government measures, which may discourage future leaders from implementing timely stay-at-home policies. These results highlight the need for authorities to leverage such insights to enhance future policies and communication strategies, reducing uncertainty and mitigating social panic.

## Introduction

### Background

The spread of the SARS-CoV-2 virus and the associated COVID-19 disease led to an extraordinary rise in uncertainty and negative emotions, resulting in significant economic and social costs [1-4]. Against this backdrop, by April 2020, most governments had implemented lockdown measures to contain the virus’s spread within their populations. Lockdown policies, or “stay-at-home” mandates, are temporary restrictions prohibiting residents from leaving their homes except for essential tasks or work in essential businesses. While recent evidence suggests that these measures are effective in slowing the spread of COVID-19 [5-7], their impact on public sentiment remains unclear. Lockdown measures may heighten public uncertainty and negative emotions by amplifying health concerns, shaping expectations of negative economic and social consequences, and triggering political backlash. Conversely, they could also signal a strong commitment to controlling the virus, reducing information asymmetry, and improving public sentiment.

### Goal of the Study

This study investigates whether lockdown measures amplify or alleviate uncertainty and negative sentiment. By proposing a nuanced measure of uncertainty and sentiment, we assess the heterogeneous effects of lockdown measures across key public concerns, including economics, health, and politics. Understanding whether the economic and social costs of lockdowns outweigh their health benefits is essential for informed policy decisions.

### Prior Work

Determining the causal effect of lockdowns on public sentiment is challenging due to simultaneity and endogeneity issues. In most countries, authorities implemented lockdown measures immediately after detecting the virus, meaning the decline in public sentiment caused by COVID-19 and the enforcement of lockdowns occurred simultaneously. Additionally, endogeneity arises because lockdowns were introduced in response to detecting COVID-19 cases.

These areas are likely the same ones where people perceive a higher risk of pandemic-related costs, resulting in lower sentiment. Given these limitations, most studies on sentiment changes during COVID-19 lockdowns provide correlational evidence [8-12], which may either overestimate or underestimate the impact of lockdowns on public emotions, potentially leading to inaccurate conclusions.

Previous studies reported increasing uncertainty and negativity during COVID-19 but did not determine the extent to which these trends were driven by restrictive measures [13,14].

Moreover, many studies examined overall changes in uncertainty and sentiment during the pandemic [15-17] or focused on specific aspects such as economic uncertainty [8,13], political polarization [18,19], or health-related negative emotions [17,20], without simultaneously analyzing multiple relevant dimensions of public discourse at the time.

Distinguishing the effects of lockdown measures across multiple topics, such as politics, economics, and health, is valuable, as their impact may vary across different dimensions.

### Our Study

To address this, we focus on the Italian lockdown of February 2020, the first in a high-income economy. This case allows for a quasi-experimental analysis, as the lockdown was initially imposed on communities where the first COVID-19 cases were detected, effectively creating a form of random assignment.

At the time of implementation, virus transmission rates were similar inside and outside the lockdown areas [21], and health and socioeconomic conditions were comparable, enabling the application of causal inference methods to assess the impact of lockdowns on public opinion. Notably, the timing of these lockdowns—before vaccines were available and with limited knowledge about the SARS-CoV-2—provides a unique opportunity to isolate and measure the direct influence of lockdown measures on public emotions, particularly sentiments and uncertainty.

We use Twitter (X) data to measure uncertainty and sentiment by applying deep learning and natural language processing techniques. To assess uncertainty, we differentiate between tweets that ask questions, express hesitation, irresolution, confusion, or anxiety, and those that seek clarity, as opposed to tweets conveying assurance and confidence. In measuring sentiment, we aim to capture emotions such as anger, disillusionment, and disapproval, which we broadly classify as negative sentiments. Additionally, we distinguish between tweets from users inside the lockdown area (the so-called red zone) and those from users outside, including neighboring cities (referred to as the “orange zone”).

This data collection is conducted both before and after the lockdown. To measure uncertainty and negative sentiments in each tweet, we fine-tune the AlBERTo (Italian BERT optimized) model [22], a natural language processing model based on the Bidirectional Encoder Representations from Transformers (BERT) framework [23], specifically designed for Italian text data. BERT efficiently contextualizes words, providing significant advantages over traditional bag-of-words approaches for measuring emotions [24,25]. It has demonstrated strong performance across various natural language processing tasks, including sentiment analysis, fake news detection, and the analysis of public opinions on Twitter/X during the COVID-19 pandemic [15,26-29]. Given the existing evidence on the accuracy of text analysis using BERT-based models, we believe that our sentiment and uncertainty measures constructed via AlBERTo reliably capture the emotions expressed in the tweets.

To better understand the impact of the lockdown on public emotions, we categorize uncertainty and sentiments into 4 main dimensions: economy, health, politics, and lockdown policy. Identifying health- and economy-related tweets is essential for analyzing how lockdown policies differently influenced perceptions of health and economic risks, as well as for investigating the health-economic trade-off [30]. The political dimension is also considered, as lockdowns could shape sentiments toward politics, affect attitudes toward incumbent politicians [31-35], or heighten political polarization [18,19]. The fourth dimension, which we refer to as policy for simplicity, captures the uncertainty and negative sentiments associated with the behavioral guidelines of the restrictions. This distinction allows us to determine whether uncertainty and negative sentiments arise from concerns about the authorities implementing the policy (political uncertainty) or from confusion regarding the policy’s specifics (uncertainty around lockdown policies). Overall, this categorization provides a clearer understanding of how public sentiment evolved in response to the COVID-19 pandemic and government actions.

We use manually curated dictionaries for each dimension to categorize the tweets and construct a document-feature matrix, enabling the identification of words associated with each dimension in the tweets.

Using a difference-in-differences (DiD) specification [36], we estimate the causal impact of the lockdown on economic, health, political, and policy-related uncertainty and negative sentiment. Causal identification is ensured as the treatment assignment—the lockdown policy—was an exogenous shock to public uncertainty and negative sentiment, independent of potential outcomes. Our findings are further validated through a placebo test and a series of robustness checks.

Our findings indicate that, in the Italian context, the lockdown had no significant effect on public concerns about the economy, suggesting that lockdown-induced negative emotions did not heighten economic worries. Economic concerns did not increase among those subjected to the lockdown, which is relevant to the ongoing debate on the economic impact of lockdowns. While stay-at-home mandates have economic consequences [37], these effects likely stem from other, more direct channels, such as business closures and activity restrictions.

By contrast, the stay-at-home mandate significantly influenced health- and politics-related emotions among individuals within the lockdown area. Users in this zone expressed higher uncertainty when discussing health and lockdown policies and displayed more negative sentiments about politics compared with those outside the lockdown area. Rather than reassuring the public about the authorities’ commitment to combating the virus, the lockdown heightened concerns about health-related risks. However, this increased uncertainty may have fostered greater awareness and compliance with containment measures, potentially leading to positive public health outcomes [38].

The rise in negative sentiments toward politics represents a significant cost that policy makers must take into account. These political costs may discourage elected officials from implementing lockdowns in future pandemics, regardless of their effectiveness in controlling virus transmission. The increased political discontent could indicate a rise in political polarization, which intensified during COVID-19 [18,19]. However, it may also reflect shifting attitudes toward the incumbent politician, for which existing evidence remains mixed [31-33,39]. The extent to which changes in political attitudes reported in the literature can be directly attributed to lockdown measures remains unclear. Our findings demonstrate that when the effect of the lockdown is causally identified and isolated from other factors influencing emotions, the policy increases negative sentiments toward politics. This shift in sentiment could lead to delays, restrictions to narrower regions, or weaker policy responses when lockdown measures are necessary.

As our study focuses on a specific case, the generalizability of our results is limited. Our findings should be interpreted as reliable causal estimates of the effect of the first Italian lockdown on the red zone units rather than as a universally applicable average across different contexts and periods. By prioritizing internal validity over external validity and providing extensive evidence supporting our empirical strategy, we isolate the direct effect of the lockdown while controlling for comoving factors, particularly exposure to COVID-19.

Our study makes a valuable and unique contribution by quantifying the direct emotional response to the extraordinary and strict public health policies of 2020. It introduces a clear methodology for analyzing geo-referenced social media data to evaluate the causal impact of policy at a local scale. This research significantly advances the current state of the art in social media analysis for studying the profound social and emotional changes brought about by the pandemic (see, eg, [40-43]).

## Methods

### Identification Strategy

Following the discovery of the first COVID-19 transmission case in the small town of Codogno, the Italian government announced a decree on February 22 imposing strict quarantine measures in Codogno and 9 neighboring municipalities in the province of Lodi (Castiglione d’Adda, Casalpusterlengo, Fombio, Maleo, Somaglia, Bertonico, Terranova dei Passerini, Castelgerundo, and San Fiorano), effective February 23, 2020 [44]. The lockdown was strictly enforced with police presence on the streets, prohibiting entry into or exit from the restricted area.

On March 8, an “orange zone” (*zona arancione*) was established, encompassing municipalities near the red zone. Lombardy and 14 other cities outside the region (Modena, Parma, Piacenza, Reggio nell’Emilia, Rimini, Pesaro e Urbino, Alessandria, Asti, Novara, Verbano-Cusio-Ossola, Vercelli, Padua, Treviso, and Venice), accounting for 16 million people, were placed under *partial* lockdown restrictions [45]. In the orange zone, residents were *invited* to avoid traveling in and out of their municipality of residence, while economic activities were allowed to continue. On March 9, these measures were extended nationwide, effectively ending the strict *red zone* restrictions in the province of Lodi [46]. A full nationwide lockdown was announced on March 22.

**Figure 1:**
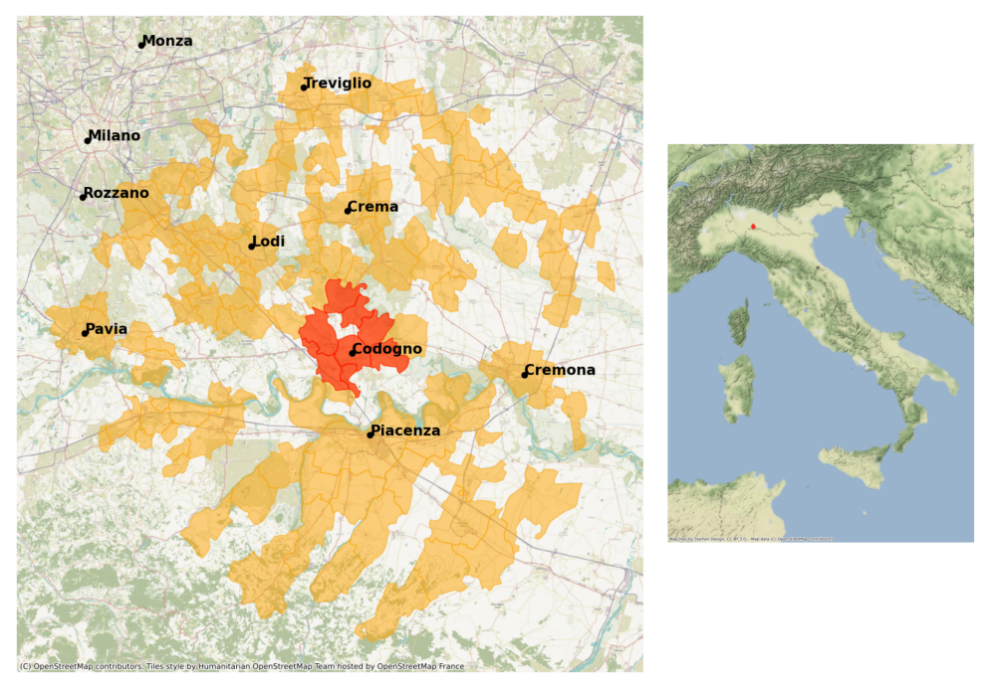
The red zone (lockdown enforced on February 23, 2020, in Lombardy, Italy) and the surrounding selected control municipalities included in the analysis are shown in orange. We found tweets from 8 out of 10 cities in the red zone. The control area consists of municipalities surrounding Codogno within a 42 km radius. The area we analyzed is homogeneous in terms of demographics, socioeconomics, and virus exposure.

We leverage the exogenous shock of the unexpected lockdown measures enacted in Italy on February 23, 2020, to assess the causal impact of lockdown restrictions on public emotions. We argue that the detection of the first European COVID-19 case in the municipality of Codogno, rather than in other nearby municipalities (Figure 1), was purely coincidental. When physicians discovered that the patient, who exhibited symptoms consistent with COVID-19, had been in contact with a friend recently returning from a business trip to China, they decided to override the existing protocol, which restricted testing to Italians and foreigners who had traveled to China [47]. As a result of this first positive case, hospitals in the Codogno area began testing individuals for COVID-19. This decision was driven solely by the identification of the first patient in Codogno.

Recent retrospective epidemiological studies on the transmission risk of coronavirus in Lombardy in February 2020 revealed that the virus had a homogeneous transmission potential across different provinces of Lombardy at the time the first case was discovered in Codogno [21]. This indicates that COVID-19 incidence was balanced between the red zone and the neighboring municipalities in the orange zone during the initial lockdown. While the red zone was subjected to strict lockdown measures, nearby locations in the orange zone were not. Consequently, individuals in the orange zone serve as a suitable control group for assessing the impact of lockdown measures on the emotions of those in the red zone.

Causal identification is achieved through the exogenous shock represented by the lockdown policy, allowing us to rule out selection bias among the units under lockdown, which are balanced and comparable to the control group. To establish a quasi-experimental approach, we designate municipalities subjected to the lockdown as the treated group, specifically the “red zone,” which was at the epicenter of the COVID-19 outbreak. The control group consists of municipalities surrounding Codogno, designated as the “orange zone.” To ensure comparability, only the closest nonurban areas to the red zone were selected.

**Figure 2:**
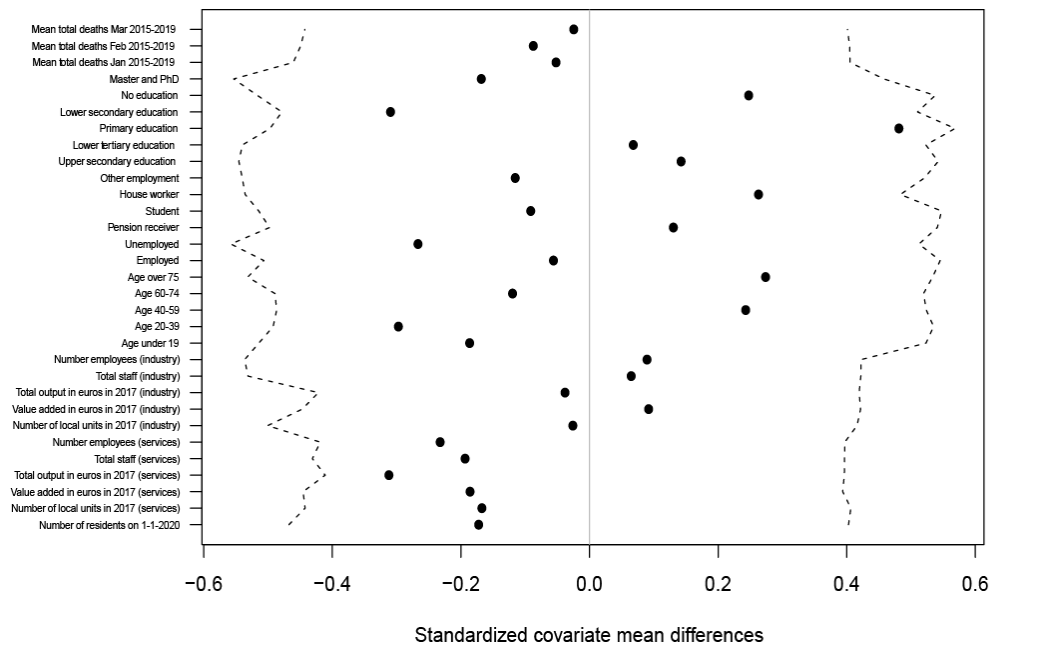
The standardized covariate mean difference was calculated along with the 7.5% and 92.5% complete randomization quantiles using 2000 permutations. Covariate balance is assessed between the red zone and the control municipalities of the orange zone, matching the user location of tweets featured in the main analysis (8 red zone and 118 orange zone municipalities). We use ISTAT data [48-52] on social, economic, and demographic characteristics, including the number of residents as of January 1, 2020; the average number of deaths in January, February, and March from 2015 to 2019; the share of residents in occupational categories, age cohorts, and education levels. Additionally, we collected data on industry and services from 2017, including total output and value added (in euros), number of employees, total staff, and local units. Missing values for industry and services are imputed using the group mean for 2 treated cities (Castelgerundo, 1473 residents, and Bertonico, 1059 residents). Similarly, missing entries for monthly average deaths are imputed for 2 control municipalities (Calendasco, 2409 residents, and Cerro all'Ambro, 5149 residents) and 1 treated city (Bertonico, 1059 residents). Before testing, we standardize covariates related to mean mortality, industry, services, and total residents.

Figure 2 [48-52] demonstrates that the complete randomization of lockdown assignment holds for municipality-level prelockdown covariates. The quantiles represent the acceptance region of our randomization test with α=.15, using the standardized covariate mean differences as the test statistic. In each iteration, the lockdown is randomly assigned to units, and the standardized mean difference is calculated. The observed standardized differences fall well within the acceptance bounds [53], confirming that complete randomization holds for all considered covariates. This finding supports the notion that the 2 areas had comparable pretreatment demographic and socioeconomic characteristics, reinforcing the validity of the random allocation.

Moreover, no other major economic or political events occurred in the area during the sample period. The region, while affected by the lockdown, is a relatively less significant hub in the local economy of Lombardia compared with urban centers such as Bergamo, Cremona, or Lodi, as shown in Table S3 in Multimedia Appendix 1. This reduces the likelihood of bias from unaccounted external events influencing public reactions.

Additionally, the influence of news outlets and media coverage on reactions in the red zone was limited, as no local media were operating within the red zone, and national media had no direct access to it. As a result, individuals inside and just outside the red zone were exposed to the same partial and limited media coverage of the lockdown policies, ensuring comparable information exposure between the 2 groups.

Our study design provides strong support for causal identification of the lockdown’s treatment effect, as the orange zone serves as a suitable counterfactual for the red zone. As the control observations share similar demographic, geographic, social, and economic characteristics and were exposed to the virus under comparable conditions, trends in overall and topic-specific uncertainty and negative sentiment should exhibit similar patterns between the treated and control groups in the absence of the lockdown intervention.

### Data Collection

For our main analysis, we collected Italian tweets before and after the lockdown, both inside and outside the red zone. Data collection took place from December 1, 2019, to March 22, 2020, using the Twitter Stream API (application programming interface). The data set includes information on user activity, user-defined locations, and the content of individual tweets.

Twitter-based indices have proven to be effective tools for understanding people’s emotional well-being during the COVID-19 pandemic [8,54]. Social media–based metrics offer a powerful means to accurately identify uncertainty and negative sentiments following major events such as COVID-19 [55]. As public reactions to crises tend to be similar among Twitter users and nonusers, emotions expressed on social media can serve as a representative proxy for the broader population’s sentiments [56].

Tweets from the municipality of Codogno and the surrounding areas are collected using the Twitter Stream API with 2 filters: Italian language and geographical location. The latter allows us to retrieve tweets with GPS coordinates within a maximum radius of 42 km from a target center—Codogno in our case. This enables us to extract tweets from the “red zone” and the “orange zone” near Codogno. We then rely on the locations self-reported by Twitter users to assign tweets to municipalities inside or outside the red zone. These self-reported locations help fine-tune user locations without relying solely on GPS coordinates to distinguish between people inside and outside the red zone, given that movement between the red and orange zones was prohibited. For an overview of Twitter’s location-based methods, see [57].

The text of self-reported user locations is cleaned and matched with ISTAT [58] data on the geographical coordinates of Italian municipalities. Additionally, the self-reported locations of tweets were manually checked to verify whether the user’s location aligned with the tweet’s content (eg, tweets indicating that Codogno users were affected by the lockdown).

We clean the text of tweets by removing URLs, hashtags, and tags and eliminate duplicate tweets—those with the same cleaned text from the same users.

On March 8, all control units (areas not yet subject to lockdown measures) entered a partial lockdown, 1 day before the nationwide expansion of partial lockdown measures on March 9, 2020. Any potential anticipation of the February 23 policy, around the time of the first detected COVID-19 transmission case in Codogno (February 20, 2020), could bias the estimate of the policy’s impact. To mitigate this, we deleted observations from February 20 to 22, 2020, and removed all tweets from March 7, 2020—the day the first extension of restrictions was announced. Finally, we removed all users who were information sources or business accounts.

As a result, we obtained a sample of 24,261 unique tweets—1567 from 8 of the 10 lockdown municipalities in the red zone and 22,694 from 118 unique locations within the orange zone. These tweets were posted by 1124 unique users (60 from the red zone and 1064 from the orange zone). Figure 1 highlights the cities with at least one active geo-referenced user account included in our analysis. We observed no tweets from 2 municipalities in the red zone: San Fiorano (1849 inhabitants) and Terranova dei Passerini (731 inhabitants). Additionally, a larger sample of tweets from across Italy (774,407 tweets) was collected during the same period using language filtering and keyword-based queries related to COVID-19. We used these tweets for our placebo analyses, detailed in the “Placebo Test” section in Multimedia Appendix 1. Using the geographical coordinates of municipalities from ISTAT [58], we identified additional unique tweets from the red zone and new municipalities in the orange zone near Codogno. These latest observations were added to the sample of tweets collected around Codogno.

The analysis is based on selected tweets from the red zone and surrounding areas. This stems from an extensive process of data processing and cleaning, which inevitably reduces the number of useful observations. In this paper, we prioritize the internal validity of our estimates over the generalizability of the results to other contexts by focusing on a single case study for credible causal inference. This approach necessitates conservative choices in selecting the subset of tweets included in the analysis to ensure the precision of both sentiment analysis and spatial mapping. Overall, the retrieved sample of users from the red and orange zones is reasonably sized, given that the geographical area covered by the policy is limited, as is its population, as shown in Table S3 in Multimedia Appendix 1.

### Ethical Considerations

The data are anonymized by removing tweets and users’ original identifiers, as well as users’ original names, and replacing them with new, deidentified user identifiers. No ethics review was sought because the study only explored publicly available data on social media and did not conduct any experiments on humans.

### Uncertainty and Sentiment Classifier

We fine-tune the pre-trained AlBERTo model [22], a deep learning natural language model trained on a large corpus of Italian tweets (~200 million, TWITA data set), to classify tweets into uncertainty and sentiment categories.

We refine the pretrained AlBERTo model in a 2-step process by manually labeling tweets for training (n=6318). The manual labels indicate degrees of uncertainty (neutral, uncertainty, and certainty) and sentiment (neutral, negative, and positive). The manually assigned labels were independently validated and verified by the authors. In the first step, we distinguish between neutral and nonneutral tweets. In the second step, we further classify nonneutral tweets as either uncertainty or certainty and as having positive or negative sentiment.

After fine-tuning the model and predicting the labels for all tweets, we obtain 2 binary variables: uncertainty and negative sentiment, assigned to each tweet in our data set. The uncertainty variable is set to 1 if the tweet expresses uncertainty—such as concern, questions, or uneasiness—and 0 otherwise, meaning the text conveys a different emotional state, such as certainty or indifference. Similarly, the negative sentiment variable is set to 1 if the tweet expresses negative feelings and 0 if it does not—that is, if it conveys positivity or neutrality. Multimedia Appendix 2 presents word clouds displaying the 100 most frequent terms in tweets classified as expressing uncertainty and negative sentiment.

### Topics Classifier

A dictionary-based classifier is used to identify topic-specific words in tweets (see Tables S1 and S2 of the “Topic Dictionaries” section in Multimedia Appendix 1 for the complete list of words used to construct the topic-specific dictionaries). We selected 4 categories likely to be influenced by the policy: health, economy, politics, and policy. For each topic, we created a binary variable indicating whether a tweet contains at least one term from the topic-specific dictionary. Overlap of topic labels within a single tweet is allowed, as tweets can cover multiple topics simultaneously. In the “Top Fifty Tweets” section in Multimedia Appendix 1, we provide a sample of tweets with the highest Shannon entropy by emotion-topic pair and discuss the performance of the machine learning classifier in identifying tweets related to each topic and emotion. Table 1 presents the number of tweets in our sample categorized as uncertainty or negative sentiment, aggregated and grouped by topic.

**Table 1:** The number of tweets classified as uncertainty and negative sentiment aggregated and grouped by topic^a^ (N=24,261).

Classification			Value, n (%)		Percentile
**Uncertainty^b^**					
	Aggregated	5270 (21.72)		89	
	Economics	369 (1.52)		99	
	Health	1063 (4.38)		97	
	Politics	301 (1.24)		99	
	Policy	304 (1.25)		99	
**Negative sentiment^b^**					
	Aggregated	8451 (34.83)		82	
	Economics	497 (2.05)		98	
	Health	1029 (4.24)		97	
	Politics	738 (3.04)		98	
	Policy	253 (1.04)		99	

^a^We have omitted the category of Leisure from the table: Uncertainty Leisure, n (%)=3498 (14.4), Negative Sentiment Leisure, n (%)=6248 (25.7).

^b^The other categories, that is, Neutral Uncertainty (Neutral Sentiment) and Certainty (Positive Sentiment), are not reported.

### Difference-in-Differences Model

To assess the causal impact of the lockdown on sentiment and uncertainty, we estimated a linear DiD model using repeated cross-sections of tweets from inside and outside the red zone before and after the February lockdown.

In the DiD model, we estimated the difference in the average change in emotion over time between the treated group (red zone) and the control group (orange zone). The estimation followed 3 steps: (1) comparing average outcomes between the treatment and control groups before the lockdown, (2) comparing average responses after the lockdown, and (3) estimating the difference. The first step accounts for preexisting differences, the second captures postlockdown differences, and the third measures the differential effect of the policy in the red zone, effectively isolating and removing time effects, common time trends, and time-invariant group differences.

For this measure to reliably estimate the treatment effect, the empirical framework must satisfy the key assumption of parallel trends, which states that emotions in the treated group would have followed the same trend as in the control group in the absence of treatment. This implies no inherent systematic differences between the treatment and control groups before the intervention. If this assumption is violated, the treatment effect may be biased. This condition is tested in the “Preexisting Trends” section in Multimedia Appendix 1. Another potential source of bias is treatment anticipation, as units might adjust their behavior in expectation of the lockdown. This is unlikely in our case, as this was the first lockdown in Europe. Another potential source of bias is spillover effects, where emotions in the red zone after policy implementation could influence those in the control group. However, because movement between the red and orange zones was prohibited, spillovers should not be relevant. We test this condition in the “SUTVA” section in Multimedia Appendix 1. Under these assumptions, the DiD estimate remains an unbiased measure of the lockdown’s causal effect on the treated units. We conducted extensive tests to validate the DiD assumptions in our quasi-experimental setting and to ensure that our statistical method was appropriate for the observational sample. This is essential for credibly interpreting the model’s estimates as the causal effect of lockdown on emotions in tweets from the red zone. The tests are summarized later in the text and detailed in the “Testing DiD Assumptions” section of Multimedia Appendix 1. As noted earlier, this case study has limitations regarding the generalizability of results and data expectations. However, by focusing on the red zone established by the February 23, 2020, policy and the selected control sample, we derive unbiased conclusions and obtain a causal estimate as if the policy had been implemented in a randomized experiment.

**Figure 3:**
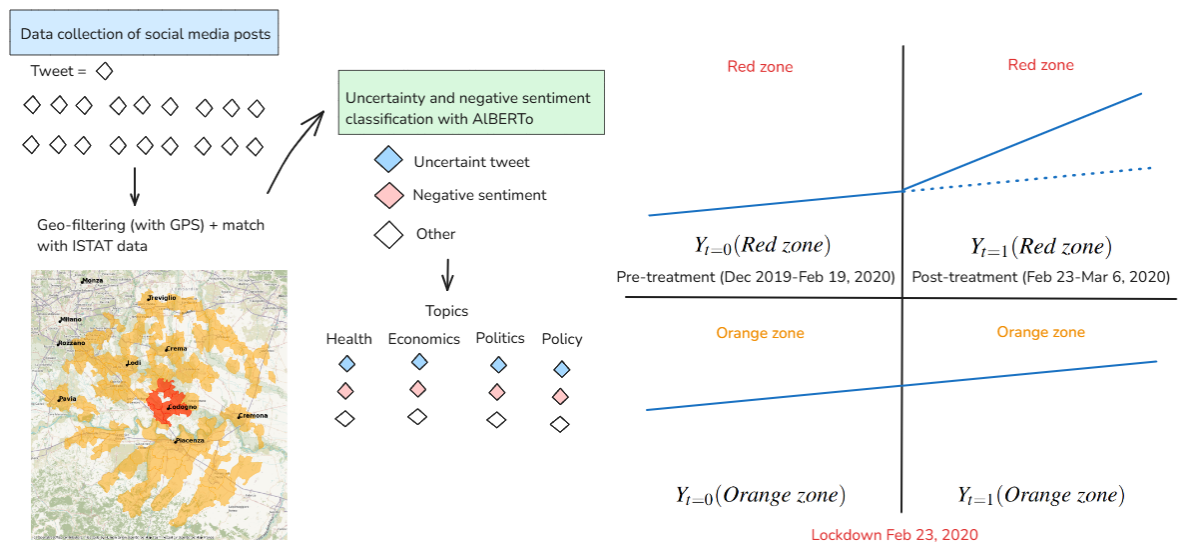
Illustrative summary of the methodology. Twitter posts are filtered using global positioning system (GPS) coordinates to select tweets posted within 42 km of Codogno. Each tweet's user location is then matched to geo-referenced ISTAT data to further filter municipalities. Sentiment analysis is performed on the retrieved set of tweets to classify the sentiment and uncertainty expressed in the text. Additionally, tweets are categorized into major topics: Health, Economics, Politics, and Policy. A difference-in-differences (DiD) approach is used to estimate the average effect of the lockdown on the red zone's emotions, measured as the difference between the average change over time (before and after 2020) in the red zone's reactions and the average change over time among the control units in the orange zone. AlBERTo: Italian BERT optimized.

We define a treatment status indicator, red zone, which equals 1 if a tweet is from the red zone and 0 otherwise. We also define a pre- and postlockdown indicator, post, where post=0 for tweets posted before the lockdown and post=1 for those posted after. As all suitable control units were eventually treated by the partial lockdown on March 9, 2020, we lost our control set. To address this, we use post=2 to identify tweets published from March 9 onward. For each tweet, we define a set of binary outcome variables indicating uncertainty and negative sentiment, either aggregated or by topic. In the DiD regression model, the coefficient of interest is post=1 × red zone=1, which represents the treatment effect—the average impact of the lockdown on the probability of observing negative reactions in tweets from the red zone. Our sample does not allow for longitudinal user analysis, as different users are present across different periods. Figure 3 provides a graphical illustration of our empirical strategy.

## Results

### Overview

The DiD model’s estimates of the COVID-19 shock’s impact on uncertainty and negative sentiment—both aggregated and by topic (economics, health, politics, and lockdown policy)—are presented in Tables 2 and 3, respectively. SEs are clustered at the municipality level (126 clusters) using the Liang-Zeger formula [59]. Model estimates with white SEs are provided in the “Model Estimates With Heteroskedasticity Robust Standard Errors” section in Multimedia Appendix 1 (also see Table S10 in Multimedia Appendix 1).

**Table 2:** Difference-in-differences regression table for uncertainty, aggregated and grouped by topics with user-level fixed effects.

Variable	Outcome 1: Aggregate	Outcome 2: Economics	Outcome 3: Health	Outcome 4: Politics	Outcome 5: Policy
	Estimate (SE^a^), 95% CI; *P* value	Estimate (SE^a^), 95% CI; *P* value	Estimate (SE^a^), 95% CI; *P* value	Estimate (SE^a^), 95% CI; *P* value	Estimate (SE^a^), 95% CI; *P* value
post=1	0.02 (0.01), –3 × 10^–6^ to 0.04; .05	–0.0006 (0.002), –0.005 to 0.003; .8	0.037 (0.005), 0.027 to 0.046; <.001	–0.0077 (0.004), -0.017 to 0.001; .09	0.01 (0.002), 0.007 to 0.014; <.001
post=2	0.022 (0.017), –0.01 to 0.056; .21	0.004 (0.002), –6 × 10^–4^ to 0.01; .08	0.042 (0.005), 0.03 to 0.052; <.001	–0.006 (0.004), –0.016 to 0.003; .19	0.01 (0.0025), 0.005 to 0.15; <.001
Red zone × post=1	0.15 (0.03), 0.09 to 0.22; <.001	0.005 (0.007), –0.01 to 0.02; .48	0.036 (0.0065), 0.023 to 0.05; <.001	0.012^b^ (0.0058), 4× 10^–4^ to 0.023; .04	0.026 (0.006), 0.015 to 0.038; <.001
Red zone × post=2	0.03 (0.05), –0.07 to 0.13; .55	–0.004 (0.01), –0.02 to 0.016; .70	–0.023 (0.01), –0.04 to –0.0037; .02	0.0138 (0.007), –0.012 to 0.015; .84	0.005 (0.011), –0.02 to 0.028; .65
Constant	0.95 (0.047), 0.85 to 1.04; 2<.001	–0.0008 (0.01), –0.02 to 0.018; .93	–0.019 (0.008), –0.035 to –0.002; .03	0.005 (0.005), –0.005 to 0.015; .34	–0.015 (0.011), –0.04 to 0.007; .18
Observations, n	24,261	24,261	24,261	24,261	24,261

^a^SEs are clustered at the municipality level.

^b^The parallel trends condition fails to be satisfied for uncertainty related to politics. See the “Robustness Checks” section and Multimedia Appendix 1 for complete analysis.

**Table 3:** Difference-in-differences regression table for negative sentiment, aggregated and grouped by topic with user-level fixed effects.

Variable	Outcome 1: Aggregate	Outcome 2: Economics	Outcome 3: Health	Outcome 4: Politics	Outcome 5: Policy
	Estimate (SE^a^), 95% CI; *P* value	Estimate (SE^a^), 95% CI; *P* value	Estimate (SE^a^), 95% CI; *P* value	Estimate (SE^a^), 95% CI; *P* value	Estimate (SE^a^), 95% CI; *P* value
post=1	–0.044 (0.022), –0.09 to –5 × 10^–4^; .047	–0.004 (0.005), –0.015 to 0.006; .42	0.036 (0.0063), 0.024 to 0.05; <.001	–0.015 (0.008), –0.03 to 0.001; .06	0.0058 (0.0017), 0.002 to 0.01; <.001
post=2	–0.056 (0.023), –0.10 to –0.01; .02	0.003 (0.0057), –0.007 to 0.015; .52	0.038 (0.0057), 0.026 to 0.05; <.001	–0.02 (0.008), –0.037 to –0.003; .02	0.0066 (0.0018), 0.003 to 0.01; <.001
Red zone × post=1	–0.072^b^ (0.027), –0.125 to –0.018; .008	–0.011 (0.0089), –0.03 to 0.006; .19	0.027 (0.021), –0.015 to 0.07; .21	0.025 (0.011), 0.003 to 0.05; .02	0.024 (0.02), –0.015 to 0.064; .22
Red zone × post=2	–0.049 (0.049), –0.146 to 0.047; .31	–0.006 (0.0126), –0.03 to 0.019; .63	0.0048 (0.022), –0.038 to 0.048; .83	0.028 (0.011), 0.006 to 0.05; .01	0.018 (0.015), –0.012 to 0.048; .23
Constant	0.105 (0.043), 0.02 to 0.19; .01	0.0024 (0.011), –0.02 to 0.024; .83	–0.042 (0.021), –0.084 to –0.001; .046	–0.008 (0.007), –0.02 to 0.01; .25	–0.024 (0.015), –0.05 to 0.005; .10
Observations, n	24,261	24,261	24,261	24,261	24,261

^a^SEs are clustered at the municipality level.

^b^The parallel trends condition fails to be satisfied for aggregated negative sentiment. See the “Robustness Checks” section and Multimedia Appendix 1 for complete analysis.

We first examine the effect of the lockdown on aggregate uncertainty. In column 1 of Table 2, the treatment effect (red zone=1 × post=1) is positive and statistically significant at .001 (b=0.15, SE 0.03, *t*_125_=4.82; *P*<.001). The estimated time trend shows no significant increase in uncertainty following the lockdown (post=1, b=0.02, SE 0.01, *t*_125_=1.98; *P*=.05) or after the implementation of partial nationwide measures (post=2, b=0.022, SE 0.017, *t*_125_=1.27; *P*=.21). Overall, the results indicate that the lockdown significantly increased aggregate uncertainty among affected individuals (b=0.15, SE 0.03, *t*_125_=4.82; *P*<.001). However, these findings may not capture the full picture, as multiple factors could influence the aggregate expression of emotions.

Column 2 of Table 2 presents the effects of the lockdown on economic uncertainty. The lockdown had no significant impact on economic uncertainty among those affected, with a small positive estimated effect (b=0.005, SE 0.007, *t*_125_=0.70; *P*=.48). This suggests that the lockdown did not substantially influence economic uncertainty.

Column 3 of Table 2 shows that the lockdown significantly increased concern about health problems among individuals in the lockdown area (b=0.036, SE 0.0065, *t*_125_=5.55; *P*<.001). As expected, after the partial nationwide measures on March 9, people in the red zone expressed significantly less uncertainty in health discussions (b=–0.023, SE 0.01, *t*_125_=–2.35; *P*=.02) as restrictions eased. However, the overall trend of health-related uncertainty increased significantly following both the local lockdown (b=0.037, SE 0.005, *t*_125_=7.50; *P*<.001) and the partial nationwide measures (b=0.042, SE 0.0052, *t*_125_=8.00; *P*<.001).

The results for political uncertainty are presented in column 4 of Table 2. The lockdown significantly increased political uncertainty in the treated area (b=0.012, SE 0.0058, *t*_125_=2.05; *P*=.04). However, this effect disappears and is no longer statistically different from 0 once all units are subjected to the same nationwide measures (red zone=1 × post=2, b=0.00138, SE 0.007, *t*_125_=0.20; *P*=.84).

Column 5 in Table 2 presents the results for uncertainty associated with the lockdown policy itself. The lockdown significantly increased concerns among those affected by the measures (b=0.026, SE 0.006, *t*_125_=4.47; *P*<.001). Additionally, the coefficients for post=1 (b=0.01, SE 0.002, *t*_125_=5.56; *P*<.001) and post=2 (b=0.01, SE 0.0025, *t*_125_=3.98; *P*<.001) indicate that uncertainty regarding the practical effects of the lockdown remained significantly higher than the baseline value.

DiD estimates of the lockdown’s effect on aggregate negative sentiment are reported in Table 3 (column 1). The lockdown significantly reduced negative sentiment among those in the red zone (b=–0.072, SE 0.027, *t*_125_=–2.67; *P*=.008). Over time, the likelihood of expressing negative sentiment decreases for both the treated and control groups, as indicated by the negative and statistically significant coefficients for post=1 (b=–0.044, SE 0.022, *t*_125_=–2.00; *P*=.047) and post=2 (b=–0.056, SE 0.023, *t*_125_=–2.45; *P*=.02). Overall, the results suggest that the lockdown significantly reduced aggregate negative sentiment among people in the red zone.

In Table 3 (column 2), we present the effect of the lockdown on negative sentiment about the economy. Although not significantly different from 0, the estimated impact on negative sentiment in economic discussions is negative (b=–0.011, SE 0.0089, *t*_125_=–1.32; *P*=.19) and relatively larger in magnitude compared with the estimated effect on economic uncertainty in Table 2 (column 2).

The regression results in Table 3 (column 3) show that the lockdown had no significant effect on negative sentiment regarding health in the lockdown area (b=0.027, SE 0.021, *t*_125_=1.27; *P*=.20). However, the overall trend indicates a significant worsening of sentiment about health issues both inside and outside the lockdown area, as reflected in the coefficients for post=1 (b=0.036, SE 0.0063, *t*_125_=5.70; *P*<.001) and post=2 (b=0.038, SE 0.0057, *t*_125_=6.57; *P*<.001).

Column 4 in Table 3 presents the effect of the lockdown on negative political sentiment. We estimate a positive and significant coefficient for both interactions, indicating that political sentiment became more negative among treated users when the first lockdown was implemented (b=0.025, SE 0.011, *t*_125_=2.33; *P*=.02) and after the nationwide measures (b=0.028, SE 0.011, *t*_125_=2.50; *P*=.01). Notably, the general trend in negative political sentiment decreased significantly following the partial national lockdown (b=–0.02, SE 0.008, *t*_125_=–2.30; *P*=.02).

In Table 3 (column 5), the red zone did not express negative sentiments about the policy significantly more often (b=0.024, SE 0.02, *t*_125_=1.22; *P*=.22). However, negative sentiment toward the policy increased across all areas, both after the first lockdown (b=0.0058, SE 0.0017, *t*_125_=3.39; *P*<.001) and after the nationwide measures (b=0.0066, SE 0.0018, *t*_125_=3.60; *P*<.001).

Overall, our results highlight an important distinction between the impact of the lockdown on economic uncertainty and economic sentiment. While economic uncertainty increased due to the spread of COVID-19 cases (as shown in [24,60]), our case study indicates that the lockdown itself did not exacerbate economic uncertainty or negative sentiment. As shown in Multimedia Appendix 2, tweets during this period were less focused on purely economic discussions: the most frequently used terms in tweets expressing uncertainty or negative sentiment were unrelated to the economy. Although the estimated coefficient for the effect of the lockdown on economic uncertainty is very close to 0 and relatively small, we cannot rule out the possibility that the lack of statistical significance for the relatively larger effect of lockdowns on negative sentiment about the economy (b=–0.011, SE 0.0089, *t*_125_=–1.32; *P*=.19) is due to the small sample size. The sample size of tweets in each topic category is provided in Figure S1 in Multimedia Appendix 1.

Instead, the lockdown affected individuals’ emotions on other topics, such as health and politics. Our results show that the lockdown increased health-related uncertainty among people in the treated area. Higher uncertainty about health issues may reflect concerns for personal safety, but it also raises public awareness of the health risks posed by the virus [38]. This suggests that the health concerns arising from the implementation of a lockdown outweigh the reassurance authorities aim to provide through lockdown measures and their commitment to containing the virus. However, our findings indicate that negative sentiment toward health issues was driven by COVID-19 itself rather than the lockdown.

The same logic applies to the impact of the lockdown on emotions expressed in discussions about the lockdown policy. Concern about the guidelines and the consequences of the policy was high among those in the red zone, but this was not accompanied by a significant change in sentiment among the treated population (b=0.024, SE 0.02, *t*_125_=1.22; *P*=.22). Sentiment in discussions about the policy worsened in both the treated and control zones after the measures were implemented, suggesting that this deterioration was likely due to COVID-19 itself rather than the lockdown.

Our results indicate that political sentiment among Twitter users in the treated area deteriorated significantly (b=0.025, SE 0.011, *t*_125_=2.33; *P*=.02). This suggests that the lockdown measures came at the cost of worsening political emotions. The increase in negative sentiment within the red zone may reflect heightened political polarization as a direct consequence of the lockdown measures, aligning with previous findings on political polarization during COVID-19 [18,19]. A rise in negative sentiment toward politics could also signal growing dissatisfaction with politicians in office. Previous research has shown that public attitudes toward politicians improved during the pandemic [35,39,61]. This aligns with the observed significant decline in the overall trend following the nationwide measures implemented on March 9 (b=–0.02, SE 0.008, *t*_125_=–2.30; *P*=.02), which came in response to a sharp increase in COVID-19 cases and deaths across the country.

In addition, the lockdown significantly worsened political uncertainty in the treated area (b=0.012, SE 0.0058, *t*_125_=2.05; *P*=.04). However, as will be explained in the next section, the estimated effect on political uncertainty is not robust to a series of validity checks on the assumptions of the DiD model.

The overall effect of the lockdown on uncertainty was positive, driven by a significant increase in uncertainty related to health (*P*<.001), policy (*P*<.001), and politics (*P*=.04). Conversely, aggregate negative sentiment in the red zone decreased. This decline was primarily due to reduced negative sentiment when discussing topics of lesser interest, mainly those related to entertainment and sports (see Table S8 in Multimedia Appendix 1). However, the estimated effect on overall negative sentiment does not hold up under most of the robustness checks we conducted, as will be explained in the next section. This reinforces the validity of the topic-based analysis in better assessing the impact of lockdowns on uncertainty and sentiment.

In general, our findings highlight the nuanced effects of lockdown policies on various emotional aspects and suggest that their impact on health, economics, and politics differs significantly. The quasi-experimental framework of our analysis allows us to distinguish between the effects of lockdown policies and broader emotional trends related to the pandemic. In the next section, we explain that the effects on aggregate negative sentiment and political uncertainty do not remain robust when subjected to a series of robustness checks.

### Robustness Checks

We conducted a battery of tests to assess the robustness of our results and summarize the key findings here.

First, we performed a series of tests to evaluate the validity of the DiD model assumptions—*parallel trends*, *absence of spillover effects*, and *no anticipation* of the treatment. To check whether the parallel trends assumption holds, we examine whether emotional reaction trends differed between the treated and control groups before the lockdown (see Figures S2 and S3 in the “Preexisting Trends” section in Multimedia Appendix 1). We find no significant differences in pretreatment trends for aggregate uncertainty, health-related uncertainty, policy uncertainty, or negative sentiment toward politics, confirming the parallel trends assumption. However, we do observe differences in pretreatment trends for aggregate negative sentiment and political uncertainty. This suggests that the DiD estimate of the lockdown’s impact on these 2 variables may be biased, as the control group does not behave as a true counterfactual, given the significant differences in prepolicy trends.

Furthermore, our findings indicate that estimates of the lockdown’s effect on political uncertainty and aggregate negative sentiment are highly sensitive to the inclusion of certain municipalities in the comparison group (see Table S9 in the “Exclusion of Territorial Administrative Units” section in Multimedia Appendix 1).

Finally, we examine the potential local spillover effects of the treatment on control units [62]. Our estimates could be biased if emotions in the control group were influenced by online reactions from the red zone. However, we argue that, in this specific context, it is reasonable to assume that red zone responses primarily affected only nearby control units, while those located beyond a certain distance remained unaffected. These distant units serve as the comparison group for the DiD estimate of the treatment effect. Our findings show that the treatment effect estimates remain consistent with the baseline DiD estimates (see Table S4 in Multimedia Appendix 1).

We also applied Benjamini-Hochberg adjusted *P* values [63] to control for the risk of false positives when testing multiple models with potentially correlated results (see Tables S5 and S6 in the “Multiple Test Adjusted *P* Values” section in Multimedia Appendix 1). The analysis confirms our findings, except for uncertainty related to politics.

As an additional robustness check, we estimate the lockdown’s effect on public emotions by comparing tweets before and after the February 23, 2020, policy across different groups: the red zone, the extended orange zone defined by the March 8, 2020, decree, and other Northern Italian locations that were later subjected to nationwide measures starting on March 9, 2020, but were never designated as a targeted priority—referred to as the “white zone” (see Figure S5 in Multimedia Appendix 1). This approach allows us to assess how spatial proximity to the lockdown area and exposure to government policy—under similar media influences—affect our findings. Pretreatment checks reveal significant demographic differences between the red zone and control groups (see Figures S6-S9 in Multimedia Appendix 1). While we rely on manually entered user locations, which are less precise than GPS coordinates, this method still enables us to distinguish tweets originating from within and outside the red zone. We randomly subsampled 20% of tweets from the extended orange zone and 50% from the white zone to ensure computational scalability in estimating the model coefficients while controlling for user-level fixed effects. Compared with the baseline DiD, the treatment effect on aggregated uncertainty, health-related uncertainty, and aggregate negative sentiment is more pronounced in the white zone but smaller in the extended orange zone. The difference increases as the distance of extended orange zone locations from the red zone grows, while it decreases as white zone locations move farther from the red zone. This suggests that differential exposure to (mild) government policies against contagion played a relevant role. Negative sentiment toward politics is higher when treated units are compared with extended orange zone locations after the policy, but the difference is significant only for those in closer proximity to the lockdown area. Additionally, we find a significant difference in economic negative sentiment between the red zone and the white zone (see Tables S11-S14 and the “DiD Estimates Varying for Spatial Proximity” section in Multimedia Appendix 1 for details).

Finally, we conducted a placebo test to examine how *anticipation* of the policy affected behavior and the estimated impact of the lockdown. Following the nationwide lockdown on March 9, we argue that municipalities with COVID-19 risk levels similar to those of the first 10 quarantined towns should not have reacted differently from those with dissimilar risk, as the nationwide measures were widely expected (see Figure S4 in the “Placebo Test” section in Multimedia Appendix 1). Using the percentage increase in monthly deaths from ISTAT as a proxy for contagion rates, we treat the lockdown as a placebo in areas where excess deaths were already high. The results indicate that the national lockdown had no significant effect on public reactions in the placebo treatment group, reinforcing the robustness of our original findings by confirming that potential anticipatory effects did not bias the estimated impact of the policy (see Table S7 in Multimedia Appendix 1).

Overall, the robustness tests support the key findings that the lockdown had significant effects on aggregate uncertainty, health-related uncertainty, policy uncertainty, and negative sentiment toward politics. However, the effects on political uncertainty and aggregate negative sentiment do not hold up across several tests.

## Discussion

### Principal Findings

Overall, our findings confirm that lockdowns come at a cost, though in an unexpected way. We found no causal impact on economic uncertainty or sentiment, suggesting that economic-related emotions were primarily driven by the rise in COVID-19 cases rather than the lockdown itself. Instead, in the red zone areas under lockdown, users were more likely to express uncertainty about health and lockdown policies, along with increased negative sentiment toward politics.

The evidence suggests that lockdowns increase, rather than reduce, uncertainty about health conditions without significantly altering sentiment in discussions on health or the management of the sanitary emergency. Our findings indicate that the deterioration of sentiment in health- and policy-related discussions is driven by COVID-19 itself rather than stay-at-home measures. Notably, lockdowns carry political costs, as affected areas experience a surge in negative political sentiment, which may deter future policy makers from implementing stay-at-home mandates to avoid political backlash.

### Comparison With Prior Work

Our contribution is 2-fold. First, we develop a new methodological framework to assess the impact of lockdown measures [31-33,39,64]. We focus on the causal effects on public emotions, an area where, to our knowledge, only correlational evidence exists [10,17,39]. Understanding how stay-at-home mandates influence public emotions is crucial for policy makers to weigh the benefits and costs of such measures.

Second, we contribute to the literature on uncertainty and sentiment, which often relies on nontraditional data such as textual analysis. Recent studies have reported particularly high levels of economic uncertainty [65] and health-related negative emotions [17,20] following the enactment of COVID-19 lockdown measures, as well as increased political polarization [18,19]. However, less is known about the specific factors driving these changes. We help bridge this knowledge gap by showing that uncertainty and negative sentiment did not increase uniformly across individuals after the lockdown but followed distinct dynamics. This is the first study to examine the effects of stay-at-home mandates on multiple dimensions of public uncertainty and sentiment.

### Limitations

It is important to note that all field experiments may suffer from a lack of external validity. Our study is no exception, as a new lockdown today could be perceived differently from the unexpected lockdown analyzed in this paper. One could argue that the public is now much more informed about the implications of a lockdown than it was in February 2020. People would adjust their strategies based on their experiences, potentially altering the policy’s impact on uncertainty and sentiment. Moreover, given the negative political sentiments such measures have generated, policy makers may now be more reluctant to implement them or, at the very least, enforce them with the same level of strictness. Nevertheless, we demonstrate that once the causal effect of the policy is effectively isolated from comoving factors and external influences, lockdowns impose political and social costs by exacerbating uncertainty and sentiment. To ensure robust and reliable causal inference, we prioritize the internal validity of our estimates over the generalizability of our results by analyzing a case study in which the policy change can be viewed as randomly allocated among groups. While it is impossible to predict how the public and policy makers will respond to a future lockdown, our findings offer valuable insights into the costs policy makers must consider before implementing new lockdown measures.

### Conclusions

Our findings suggest that communication surrounding lockdown measures may have been unclear, fostering resentment toward the political class among those affected and failing to generate explicit support for the government’s plans. Policy makers must enhance communication to reduce uncertainty and negative sentiment [66]. These findings underscore the need for authorities to refine future policies and communication strategies to mitigate uncertainty and social panic.

## Appendix

Multimedia Appendix 1 Supplementary analysis and results.

Multimedia Appendix 2 Word clouds of the 100 most frequent terms in tweets classified as (A) aggregated uncertainty and (B) negative sentiment. In our thematic categories, the most prominent terms in A relate to health (eg, coronavirus, virus, cases [casi], deaths [morti], patient [paziente], asymptomatic [asintomatico]), politics (eg, Europe [Europa], parties [partito]), and lockdown policy (eg, supplies/food [viveri necessari], closures [chiuderlo]). In panel B, the most frequently occurring words belong to the health category (eg, coronavirus, deaths [morti], ambulances [ambulanze]) and the lockdown policy category (eg, closures [chiuderlo], provisions/food supplies [viveri necessari]). The complete set of words belonging to each thematic category is listed and translated in Tables S1 and S2 in Multimedia Appendix 1.

## Abbreviations

AlBERTo: Italian BERT optimized
API: application programming interface
BERT: Bidirectional Encoder Representations from Transformers
DiD: difference-in-differences

## Data Availability

The data and code underlying this article are available in Zenodo and can be accessed at [67].
