# Supplementary material for: The Impact of Stay-At-Home Mandates on Uncertainty and Sentiments: Quasi-Experimental Study
Source: J Med Internet Res. 2025 Mar 4;27:e64667. doi: 10.2196/64667 (PMC11920662; doi:10.2196/64667)
Supplement: Multimedia Appendix 1 [file jmir_v27i1e64667_app1.pdf]

Multimedia Appendix 1

A Topic Dictionaries

We rely on a dictionary-based classifier to classify tweets into topic. For each topic, we define a list of topic-specific commonly used words. The classifier then counts the number of terms used in each tweet that belongs to each list. The complete list of words used to construct the topic-specific dictionaries are reported in Tables S1 (in Italian) and S2 (in English).

We create one dummy variable for each dictionary, that equals 1 when the tweet contains at least one term from the dictionaries and 0 otherwise.

In Figure S1, we report the total number of tweets retrieved for each topic, as well as the number of tweets that are shared between topical categories.

In the process of extracting topics via dictionary-based classifiers, we clean the tweets and remove all URLs, tagging, and punctuation. For hashtags, we remove the hashtags, but keep the tagged word.

Economics
economia, economic*, soldi, investiment*, banc*, finanz*, disoccupa*, bancarott*, imprenditor*, impres*, lavoro, bonus, commerci*, gestione, piano, sostegno, crisi, iva, dipendent*, fiscal*, coldiretti, agevolazioni, contribut*, reddito, salari*, confedilizia, confindustria, cgil, professionist*, negoz*, euro, mutuo, mutui, tasse, tassa, tassazione, tassat*, evasione, fisco, sindacat*, inps, credit*, prestit*, stipendio, deficit, lavorator*, produzion*, produttiv*, aziend*, client*, soci, salone, ristorant*, smart-working, smart working, commercial*, supermercat*, spes*, mercat*, turist*, turismo, licenzia* fiera, fiere, cassa integrazione, lavorare, lavorare a casa.
**Health**
coronavirus, virus, contag*, covid, tampon*, mascher*, casi, quaranten*, mort*, ospedal*, malat*, malatti*, sanità, sanitari*, medic*, medicina, infermier*, positiv*, farmaci*, kn95, terapi*, terapia intensiva, terapie intensive, sars, sars-cov-2, paziente zero, pazient*, infett*, salute,decess*, influenza, peste, sanita, guarit*, guarigion*, ammarlarsi, ammalarci, ammalat*, ammalare, covid19, croce rossa, epidemiolog*, oms, febbre, asintomatic*, rianimazione, epidemia, respiratori, ricoverat*, portatore sano.
**Politics**
politic*, govern*, italiaviva, salvini, renzi, conte, meloni, presidente, lega, ministro, sindaco, decreto, legge, movimento5stelle, mattarella, segretari*, legislativo, parlament*, giunta, assessor*, ue, politic*, profughi, pd, ong, sinistra, migranti, democrazia, democratic*, partito, partiti, sardine, dimettiti, dimission*, fascismo, fascist*, 5s, protesta, contedimettiti, nazismo, nazist*, destra, casta, m5s.
**Policy**
chius*, sospes*, cancellat*, limitazion*, annullat*, chiud*, sospension*, isolat*, isolamento, rinviat*, scorte, viveri necessari, zona rossa, zona arancione, luoghi di aggregazione, distanza, restrizion*, controlli, posto di blocco, posti di blocco, spostamenti, autocertificazione.

Table S1: Dictionaries for classification into *Topics* in Italian

Economics
economy, economic*, money, investment*, bank*, financ*, unemployed, bankruptcy, entrepreneur*, business*, work, bonus, trade, management, plan, support, crisis, VAT, employee*, fiscal*, coldiretti, subsidies, contribution*, income, wage*, confedilizia, confindustria, cgil, professional*, shop*, euro, mortgage, mortgages, taxes, tax, taxation, evasion, taxman, trade union*, INPS, credit*, loan*, salary, deficit, worker*, production, productive*, company*, client*, partners, salon, restaurant*, smart-working, smart working, commercial*, supermarket*, expens*, market*, turist*, turism, licens*, fair, fairs, layoffs, work, work at home.
**Health**
coronavirus, virus, contagion*, covid, swab*, mask*, cases, quarantin*, death*, hospital*, sick*, sickness*, health, sanitary*, medic*, medicine, nurse*, positive*, drugs*, kn95, therapy*, intensive care, ICU, sars, sars-cov-2, patient zero, patient*, infected*, death*, flu, plague, sanitation, heal*, ill*, recover*, covid19, red cross, epidemiolog*, oms, fever, asymptomatic, resuscitation, epidemic*, respiratory, hospitaliz*, carrier*.
**Politics**
politic*, govern*, italiaviva, salvini, renzi, conte, meloni, president, lega, minister, mayor, decree, law, 5stelle movement, mattarella, secretaries*, legislative, parliament*, assessor*, eu, refugees, pd, ngo, left, migrants, democracy, democratic*, party, parties, sardines, resign, resign*, fascism, fascist*, 5s, protest, conteresign, nazism, nazist*, right, casts, m5s.
**Policy**
clos*, suspend*, cancel*, restrict*, isolated, isolation, postpon*, supplies, necessary food, red zone, orange zone, meeting places, distanc*, controls, checkpoint, checkpoints, travel, self-certification.

Table S2: Dictionaries for classification into *Topics* in English

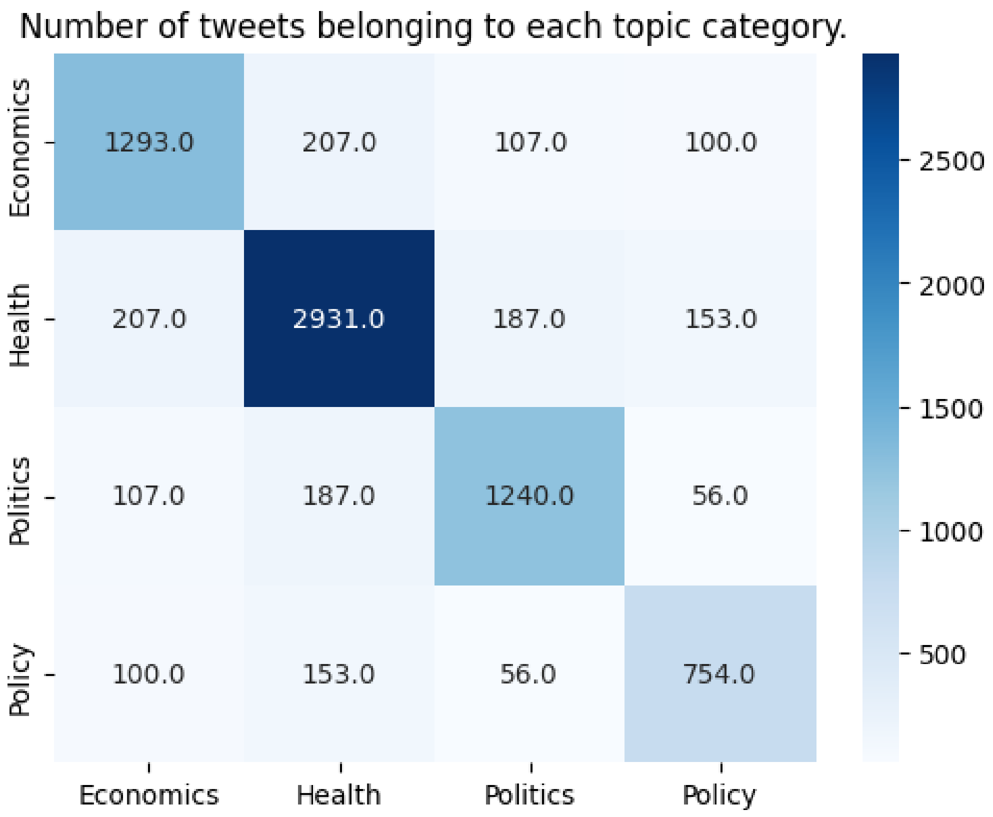

Figure S1: Number of tweets belonging to each topic category. The entries on the main diagonal refer to the total count of tweets labelled as (i) economics, (ii) health, (iii) politics, (iv) policy. The remaining entries report the number of tweets that are common between pairs of topics.

B Top Fifty Tweets

In this section, we provide some examples of tweets that presented the highest Shannon entropy scores for each emotion-topic pair. For each pair, we select five examples for illustration. We used Google Translate to translate each tweet from Italian to English and adjusted the translation whenever needed. We report in parenthesis and in bold the Shannon entropy score of each tweet. When necessary, we add some notes in square brackets to ease the interpretation of the text.

The classifier performs well in capturing sentiments, with a few minor drawbacks. The classifier proves successful in identifying the topics and detecting negative sentiments, even when the text is sarcastic. For instance, in the second tweet among health negative sentiments, the user sends 'big congratulations' to the prime minister for his 'perfect checks' on COVID-19 cases. Despite the use of these words, which out of context

are considered positive, the sentiment of the tweet is identified as negative. Negative sentiments in tweets encompass a range of negative emotions, including fear and anger.

There is some inevitable overlap across topics, which however does not denote a mistake by the classifier, but stems from the actual overlap of topics in the text. For instance, some tweets with the highest entropy in terms of economic negative sentiments are also related to politics, as they blame 'Italian politics' for the 'incalculable economic damage' they created. The same happens for some politics-related tweets, where the Prime Minister is sarcastically praised for his 'perfect checks' in light of the rising risks of coronavirus infections. Notably, this tweet appears in the top 50 of the categories 'politics' and 'health'.

Economic Uncertainty:

- Very little money is saved and incalculable damage is done [**.97**]
- It will be the beginning of our financial monetary crisis that we will not be able to sustain which will force us to leave the URL [**.95**]
- It seems so. We await technical details on closing all commercial activities except for public utilities [**.92**]
- But are the migrant gentlemen in front of the supermarkets immune from the March decree or do they all have a self-certification? [**.84**]
- #fightcoronavirus hashtag #cremona don't come to the bank better do everything online - appeal of the bank unions URL [**.78**]

Economics Negative Sentiments:

- I work in psychiatry I can tell you that in the red zone it's crazy as they say [**0.72**]
- The lightness of #italianpolitics in facing #covid is provoking incalculable economic damage [**.53**]
- Meanwhile those who are part of the productive sector must not stay at home but go and get infected for the good of the capital [**.45**]
- #Istayhome ah no I can't work in insurances or banks. #factory i don't have the right to #stayhome it's a shame [**.48**]

- you ruined italy: all the Italian sectors from the craftsman trader to tourism etc. [**.22**]

Health Uncertainty:

- I think that in November in the red zone there will be a demographic explosion of positive covid obviously [**.99**]
- Coronavirus Amendola [Minister of European Affairs]: it is possible that the EU [European Union] will give us more budget flexibility [**.98**]
- By now we know who Salvini [leader of the right-wing political party] is. The problem is how much is true about this virus [**.98**]
- For the first time since the beginning of the emergency I have news of a person in intensive care and another who died [**.95**]
- if we healthcare workers wore [face masks] as he did we would make a massacre [**.93**]

Health Negative Sentiments:

- you wake up and a little anxiety comes over you early in the morning #coronavirus [**.99**]
- #Conte [Italy's prime minister] #wakeup the #coronavirus advances and reached Lodi [one of the main cities in the red zone] big congratulations to you, perfect checks [**.77**]
- more than a month to declare we are ready everything is under control and then the infection comes [**.72**]
- My mom is in the hospital and they don't have face masks #coronavirus #covid #italy and they made everyone swab [**.71**]
- number of infections from #coronavirus are on the rise but in #Piacenza there is no outbreak [**.66**]

Politics Uncertainty:

- If we all closed the borders we would all be [considered] fascists [**.99**]
- I thought that after Napolitano [ex President of the Republic] we had hit rock bottom but Mattarella [current President of the Republic] never missed an opportunity to do worse [**.99**]
- By now we know who Salvini [leader of the right-wing political party] is. The problem is how much is true about this virus [**.98**]
- I can't get over that someone really believes that the EU will help Italy [**.87**]
- OMG honorable [Member of Parliament] you who called your prime minister a criminal? This is not very credible, right? [.82]

Politics Negative Sentiments:

- #Conte [Italy's prime minister] #wakeup the #coronavirus advances and reached Lodi [one of the main cities in the red zone] big congratulations to you, perfect checks [**.77**]
- The lightness of #italianpolitics in facing #covid is provoking incalculable economic damage [**.53**]
- #Conte shifts the blame of #covid contagions to the hospital of Codogno where they have worked hard and they keep working hard with their shifts [**.41**]
- #coronavirus taught us that the #nationalhealthservice must be refounded and that the #TAV [high speed train project that has been subject to heated politically debates] is useless and #Conte is useless [**.32**]
- Intensive care doctors from several hospitals have called for the silence of politicians [**.26**]

Policy Uncertainty:

- the problem is that there is a fear of widening the red zone [**.99**]
- It's absurd that the TV information we have in the red zone is the same as the rest of Italy [**.98**]

- Hello Valerio I'm a nurse in the red zone. [Here] the measurements are disproportionate [**.94**]
- I am biased but yes we are apparently closed but all of a sudden you will find yourself at the [same] table with us [i.e., in the same situation] [**.92**]
- it is yet to be clarified if the match will be played behind closed doors [**.84**]

Policy Negative Sentiments:

- I work in psychiatry I can tell you that in the red zone there is madness as they say [**.72**]
- it is yet to be clarified if the match will be played behind closed doors [**.64**]
- but I ask how can you really close everything [?] what a desolation all places ['locali', meaning bars, restaurants...] closed #coronavirus #italy #cremona [**.61**]
- the postponement of Serie A matches scheduled behind closed doors is simply shameful [**.22**]
- it must be that the closed doors mandate in Piedmont [region in North West Italy] is not valid and there are no infected people [**.20**]

C Descriptive Socio-economic and Demographic Indices of the Red Zone

As shown in Table S3 reporting industrial and services indices on output, units, value added and employed labor force by total population, the red zone has limited relevance in the regional economy when compared to other nearby urban centers in Lombardia (Italy) which instead were not affected by the policy. Moreover, our strategy privileges internal validity over external validity; our conservative and extensive process of cleaning the collection of tweets to ensure robust tweet allocation between different areas, and reliable sentiment analysis allows us to enact our identification strategy and to efficiently isolate reactions in tweets from inside and outside the red zone. As our sample features a relatively small set of 62 users, our collected sample of over 1 500 cleaned and reliable

tweets from the lockdown area – which is the unit level of analysis – is nevertheless a reasonable result, as the population from the red zone is very restricted, as well as its geographical extension – see Table S3.

Location	$Units_{Serv}$	$Units_{Ind}$	Res. Pop.	$Value_{Serv}$	$Output_{Serv}$	$Rate(Emp)_{Serv}$	$Value_{Ind}$	$Output_{Ind}$	$Rate(Emp)_{Ind}$
Bergamo	157.42	33.17	2987.87	6085.68	24693.52	0.09	2388.31	9453.17	0.03
Brescia	95.61	18.13	2184.02	4395.62	23143.12	0.07	3977.05	23851.92	0.05
Cremona	31.79	9.49	1010.40	2639.37	12025.10	0.04	7007.91	33190.73	0.05
Lodi	35.96	8.41	1083.01	3646.11	11605.07	0.04	567.06	2808.02	0.01
Milano	384.09	73.30	7566.37	12369.84	57356.25	0.12	2538.59	12046.12	0.03
Pavia	39.82	7.72	1131.43	2152.99	10149.71	0.04	556.40	1822.39	0.01
Bertonico	0.82	NaN	51.32	881.94	1707.52	0.02	NaN	NaN	NaN
Casalpusterlengo	14.02	6.60	594.03	1039.61	4078.39	0.02	1986.32	6773.12	0.02
Castelgerundo	NaN	NaN	75.04	NaN	NaN	NaN	NaN	NaN	NaN
Castiglionedadda	7.01	3.08	346.84	972.81	3840.00	0.01	4673.97	14414.59	0.06
Codogno	21.66	8.62	744.99	2924.82	6372.88	0.03	7545.03	23114.22	0.09
Fombio	5.14	3.65	306.08	1596.77	4856.80	0.02	5273.86	24937.72	0.08
Maleo	2.47	1.71	151.69	1035.85	2768.28	0.02	518.99	1620.10	0.01
Somaglia	3.27	2.50	185.73	2504.82	11128.20	0.04	3095.74	13484.12	0.05

Table S3: Istat demographic and economic indices for municipalities in the red zone. Two out of the ten municipalities in the red zone – San Fiorano (1,849 inhabitants) and Terranova dei Passerini (731 inhabitants)– are not coved by Istat. Local Units (*Units*) and Residential Population at January 1, 2020 (*Res. Pop.*) are adjusted by total area in square kilometers; the remaining indicators are divided by the municipality's total residents. The locations of the red zone do not represent a major economic hub of Northern Italy: the number of residents does not exceed the 16 thousands, and industrial output and labor force is contained with respect to locations in the area (Bergamo, Brescia, Cremona, Lodi, Milano, Pavia). With the subscript *Ind* (*Serv*), we indicate statistics for the industrial sector (services). Output and Value Added (*Value*) are measured in Euros; $Rate(Emp)_{Ind}$ ($Rate(Emp)_{Serv}$) indicates the number of employees in the industrial (services) sector in a given municipality over the municipality's total number of residents.

D Testing DiD Assumptions

D.1 Pre-existing trends

The validity of a DID model rests on the key identification assumption of *parallel trends*. We follow [1] to check for significant differences in pre-treatment trends between the red zone and the control units.

In Figures S2 and S3, we see that the magnitude and significance of coefficients are in line with previous findings for aggregated uncertainty and uncertainty towards health and the policy, and negative sentiment related to politics, as the difference in pre-treatment trends is not statistically significant. On the other hand, we reject the hypothesis of parallel pre-treatment trends for aggregated negative sentiment and uncertainty towards politics.

Although it is common practice to perform statistical hypothesis testing of the differences in pre-treatment trends between treated and control groups, the assumption still fundamentally relies on unobservable quantities, that is, the outcome of the treatment group in absence of the treatment. Not rejecting the parallel trends hypothesis is neither a necessary nor a sufficient condition of the DID model, which still remains untestable and based on unobservable counterfactual quantities. Throughout the paper, we provide a logical explanation of why the parallel trend assumption should hold between the red zone units and the selected controls, as the conditions for parallel counterfactual trends hold only by the logical reasoning underpinning the empirical design [2].

D.2 SUTVA

An implicit assumption of a DiD model is the *Stable Unit Treatment Values Assumption* (SUTVA), which states that the potential outcome of a unit cannot depend on the treatment assignment or the potential outcome of another unit. A violation of the SUTVA means that we have multiple treatment effects, depending on who is treated [3].

Preventing units from interacting with each other is often difficult, if not impossible [4], especially as we are dealing with social media. In our model, there could be a violation of the SUTVA as one could potentially argue that the outcome of the control group is positively correlated with the reaction of those under lockdown through social and virtual connections.

One can try to disentangle the spillovers from the treatment effect by making assumptions on the exposure effects. In our setting, it seems reasonable to assume that spillovers of the treatment will affect nearby control units, while exposure to externalities of the treatment could be ignored for units located above a certain distance from the lockdown area. Under the assumption that spillovers are local, SUTVA will be violated only for units within a certain distance from the treated units [5].

Following [5], we create dummy identifiers for *distance from the red zone*, a set of *rings* surrounding the treatment units with increasing distance, and interact them with the treatment indicator. Our data features units coming from a small geographical area surrounding the red zone, encompassing all red zone locations and orange zone municipalities in the surrounding area. Here we assume that units located at more than 30 km from the red zone are not affected by spillovers. Then we estimate the effect of the

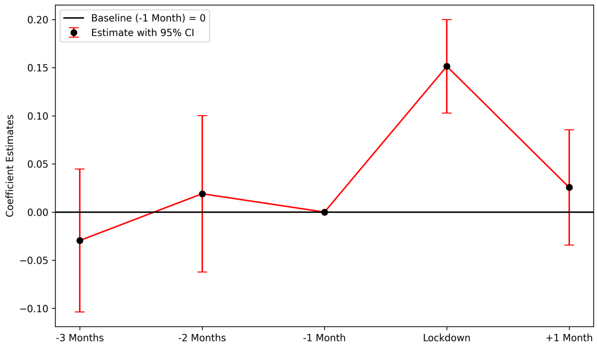

(a) Aggregated

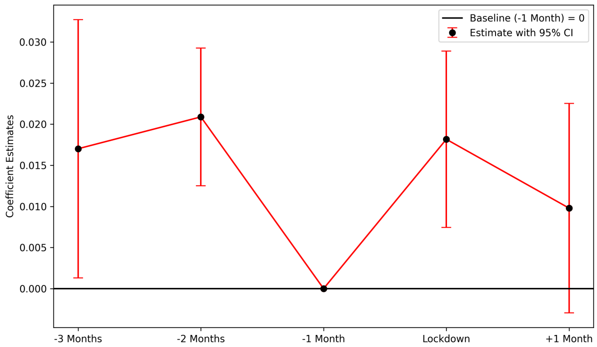

(b) Economics

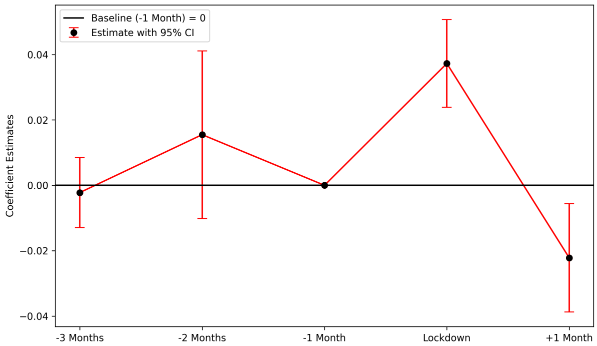

(c) Health

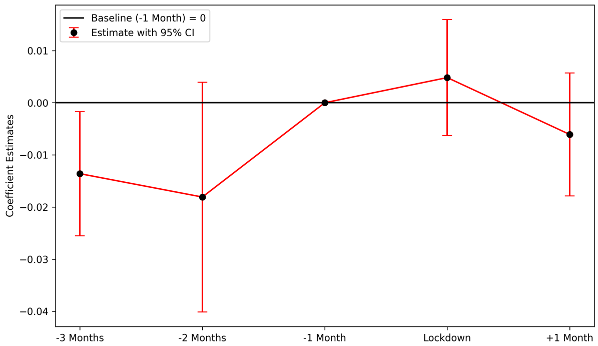

(d) Politics

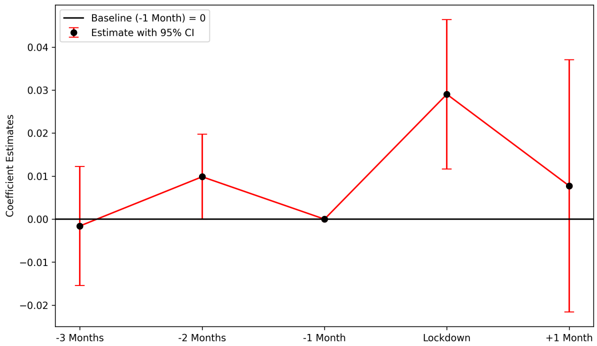

(e) Policy

Figure S2: Coefficient Estimates and Confidence Intervals (% 95) of interaction between the treatment variable and time dummies. The dependent variable is Share of tweets classified as *Uncertainty*, aggregated and grouped by Topic. The baseline period starts from the 1rst of February and ends on the 19th of February — Month -1. The lockdown period goes from February 23 to March 6, 2020; data collection stops on March 22.

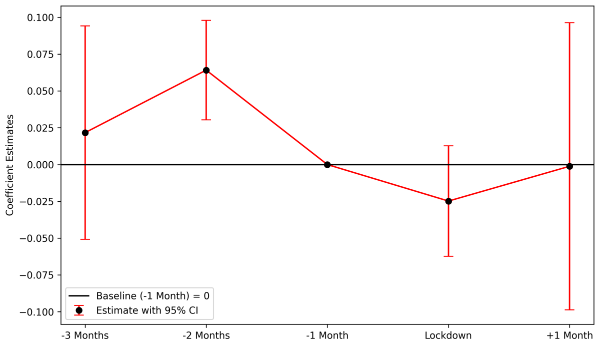

(a) Aggregated

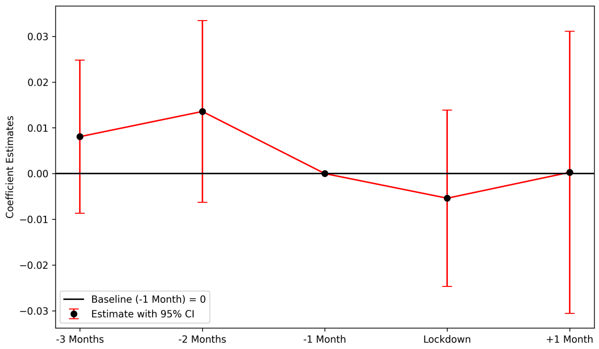

(b) Economics

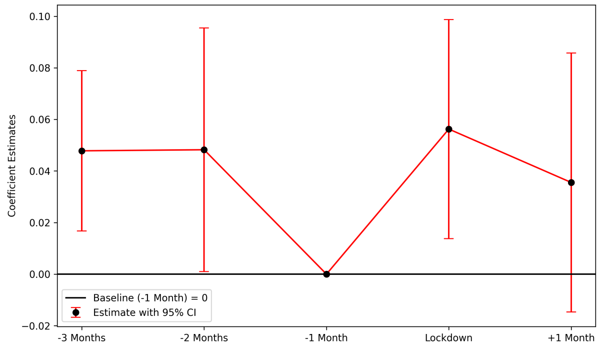

(c) Health

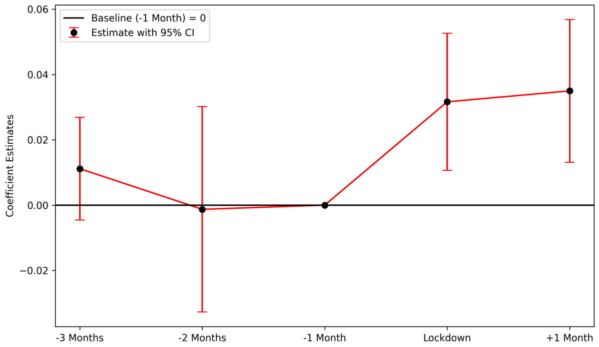

(d) Politics

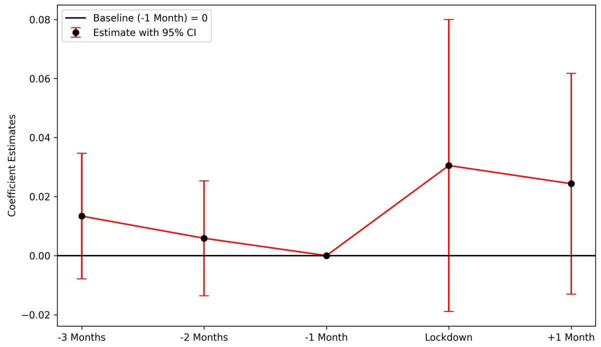

(e) Policy

Figure S3: Coefficient Estimates and Confidence Intervals (% 95) of interaction between the treatment variable and time dummies. The dependent variable is Share of tweets classified *Negative Sentiment* aggregated and grouped by Topic. The baseline period starts from the 1rst of February and ends on the 19th of February — Month -1. The lockdown period goes from February 23 to March 6, 2020; data collection stops on March 22.

lockdown comparing changes in the red zone with units above 30 kms, while controlling for the average spillover on closer control units – located between zero and 20 kms from the red zone, and between 20 and 30 kms.

In Table S4, we find that the results are in line with the baseline estimates of the effect of the local lockdown of February 23, 2020. We retrieve a significant estimate of the effect of interest (redzone=1 × post=1) on aggregated uncertainty and negative sentiments, uncertainty related to health and the policy, as well as uncertainty and negative sentiments towards politics.

	Uncertainty					Negative Sentiment				
	(1) Aggregate	(2) Economics	(3) Health	(4) Politics	(5) Policy	(6) Aggregate	(7) Economics	(8) Health	(9) Politics	(10) Policy
post=1	0.0202	-0.0000794	0.0369	-0.00781	0.0110	-0.0430	-0.00388	0.0362	-0.0159	0.00603
	(0.0101)	(0.00213)	(0.00486)	(0.00455)	(0.00194)	(0.0227)	(0.00521)	(0.00643)	(0.00826)	(0.00165)
post=2	0.0220	0.00502	0.0421	-0.00638	0.0103	-0.0549	0.00402	0.0380	-0.0205	0.00686
	(0.0172)	(0.00250)	(0.00519)	(0.00487)	(0.00248)	(0.0235)	(0.00561)	(0.00579)	(0.00876)	(0.00180)
redzone=1 × post=1	0.154	0.00472	0.0360	0.0121	0.0263	-0.0733	-0.0121	0.0268	0.0260	0.0244
	(0.0321)	(0.00740)	(0.00644)	(0.00589)	(0.00594)	(0.0274)	(0.00888)	(0.0212)	(0.0110)	(0.0201)
redzone=1 × post=2	0.0300	-0.00421	-0.0233	0.00145	0.00506	-0.0508	-0.00639	0.00468	0.0288	0.0179
	(0.0504)	(0.00985)	(0.00981)	(0.00707)	(0.0117)	(0.0490)	(0.0126)	(0.0219)	(0.0114)	(0.0151)
$\eta_{(0,20]}$	0.0830	0.0363	-0.331	-0.00768	0.0232	-0.274	0.0237	-0.333	-0.0299	0.0179
	(0.0163)	(0.00310)	(0.0115)	(0.00422)	(0.00310)	(0.0214)	(0.00539)	(0.00763)	(0.00692)	(0.00162)
$\eta_{(20,30]}$	0.0649	-0.00510	-0.339	-0.00143	0.000682	-0.388	-0.00790	-0.335	0.00464	-0.000828
	(0.00876)	(0.00148)	(0.00505)	(0.00199)	(0.00225)	(0.00791)	(0.00241)	(0.00308)	(0.00250)	(0.00105)
Constant	0.948	-0.000804	-0.0188	0.00494	-0.0154	0.106	0.00237	-0.0427	-0.00831	-0.0247
	(0.0474)	(0.00953)	(0.00832)	(0.00513)	(0.0115)	(0.0430)	(0.0113)	(0.0211)	(0.00724)	(0.0150)
Observations	24261	24261	24261	24261	24261	24261	24261	24261	24261	24261
Adjusted R^2	0.047	0.038	0.130	0.025	0.042	0.098	0.035	0.107	0.045	0.058
Clustered SE	Yes	Yes	Yes	Yes	Yes	Yes	Yes	Yes	Yes	Yes

Standard errors in parentheses

Table S4: Estimates of spillover effect and total treatment effect for *Uncertainty* and *Negative Sentiment*, with clustered standard errors (in parenthesis). The coefficient η_d return the average spillover effect on the control units within distance $d := \{(0, 20], (20, 30]\}$ in the post-treatment period.

E Multiple Test Adjusted P-values

We report the Benjamini-Hochberg adjusted p-values [6] for multiple hypothesis testing for false discovery rate. For aggregated uncertainty, we jointly test the p-values of the model in equation (1) of the main text with regressions on two binary variables representing the correlated categories of *neutral* uncertainty and *certainty*; for negative sentiment, we jointly test the p-values with regressions on *positive* and *neutral* sentiment. Considering uncertainty and negative sentiment grouped by possible topics, the p-values of the four topic-related independent regressions are adjusted together. In Tables S5 and S6,

Uncertainty	Aggregate	Aggregate	Economics	Economics	Health	Health	Politics	Politics	Policy	Policy
	P(>|t|)	BH	P(>|t|)	BH	P(>|t|)	BH	P(>|t|)	BH	P(>|t|)	BH
$post = 1$	0.0478	0.0718	0.8	0.8448	0	0	0.086	0.1376	0	0
$post = 2$	0.2043	0.2452	0.0812	0.1376	0	0	0.1912	0.2781	1e-04	2e-04
$redzone = 1 \times post = 1$	0	0	0.4832	0.6443	0	0	0.0399	0.0798	0	0
$redzone = 1 \times post = 2$	0.5492	0.5492	0.7023	0.8026	0.0187	0.0428	0.8448	0.8448	0.6525	0.8026

Table S5: Benjamini-Hochberg Adjusted pvalues for *Uncertainty*, aggregated and grouped by topics with user fixed effects. P(>|t|) reports the unadjusted p-values; BH the adjusted p-values.

Negative Sentiment	Aggregate	Aggregate	Economics	Economics	Health	Health	Politics	Politics	Policy	Policy
	P(>|t|)	BH	P(>|t|)	BH	P(>|t|)	BH	P(>|t|)	BH	P(>|t|)	BH
$post = 1$	0.0451	0.0542	0.4176	0.5139	0	0	0.0606	0.1212	7e-04	0.0028
$post = 2$	0.0143	0.0271	0.5155	0.5891	0	0	0.0215	0.0491	3e-04	0.0017
$redzone = 1 \times post = 1$	0.0075	0.018	0.1873	0.3087	0.2053	0.3087	0.0197	0.0491	0.2211	0.3087
$redzone = 1 \times post = 2$	0.3102	0.3384	0.6316	0.6737	0.8252	0.8252	0.0123	0.0392	0.2315	0.3087

Table S6: Benjamini-Hochberg Adjusted pvalues for *Negative Sentiment*, aggregated and grouped by topics with user fixed effects. P(>|t|) reports the unadjusted p-values; BH the adjusted p-values.

we confirm all main findings of the baseline estimation as the treatment effect (*red zone* x *post* = 1) retains its statistical significance on aggregated uncertainty and negative sentiment, uncertainty towards health and the policy guidelines, and negative sentiment towards politics. The effect is no longer significantly impacting the treated group's uncertainty towards politics. This indicates that the original p-value of the policy effect on public reaction was indeed inflated by correlations among the regression models, leading to an incorrect inference.

F Placebo Test

We perform a *placebo* test to test whether we can retrieve the effects of the lockdown when the implementation of quarantine measures does not come as *unexpected* by the public.

We focus on the partial national lockdown of March 9 and argue that in those municipalities where the exposure to the risk of the virus is *similar* to the first 10 municipalities under quarantine, there should not be any particular different reaction compared to municipalities with a totally dissimilar exposure to the risk, after the first national lockdown, since the announcement of the nation-wide measures were easily anticipated and expected.

We take the percentage increase of total deaths at the monthly and municipality level available on the Istat archives [7] as a proxy variable for the underlying rate of contagion,

or *exposure to risk* of contagion. Excess death rates represent a suitable proxy for the underlying rate of contagion, especially since mass testing for COVID-19 was still not a possibility at the time of the policy and we cannot directly observe the rate of contagion. In order words, the placebo test analogy considers the national lockdown policy as a *placebo* treatment on locations in which the exposure to the risk of COVID-19 in terms of excess deaths was already high, with values comparable to those registered in the red zone. Because it is a *placebo test*, the policy itself should not change the overall behavior of people. This enables us to test how anticipating the policy changes the estimates of the impact of the policy, as people adjust their behavior knowing that the change is coming.

For each municipality, the percentage increase over a given month in 2020 with respect to the average for 2015-2019 is computed as the total number of deaths in that month minus the mean of the total number of deaths in the same month over the years 2015-2019, divided by the mean of the total deaths of that month over the period 2015-2019:

$$\Delta(month) = \frac{DeathToll(month)_{2020} - MeanDeathToll(month)_{2015-2019}}{MeanDeathToll(month)_{2015-2019}}$$

For instance, if we take the month of January 2020, we compute the total number of deaths in January 2020 minus the mean of the total number of deaths of January over the years 2015-2019, divided the mean of the total deaths of January over 2015-2019.

A large sample of tweets from all over Italy (774,407 tweets) was collected during February and March 2020, using language filtering and keyword-based queries related to COVID-19. Using geographical coordinates of municipalities retrieved via Istat [8], we are able to identify tweets geo-located in 1,013 municipalities from the North of Italy only (72,635 observations), and exclude those municipalities used as controls for the Diff-In-Diff models. Because we wish to estimate the effect of the national lockdown of March 9 on placebo treated and control groups of tweets, we drop all observations from March 8, as some municipalities enter quarantine one day before the national lockdown. We also remove March 7, the day of the announcement of the one-day lockdown for 14 provinces in Northern Italy.

For each municipality, we consider the percentage increase of deaths of January and February 2020. Each location is represented by a two-dimensional vector. We compute the Euclidean distance between a municipality's vector and the January and February percentage increase of deaths averaged over the ten locations under the first lockdown. We

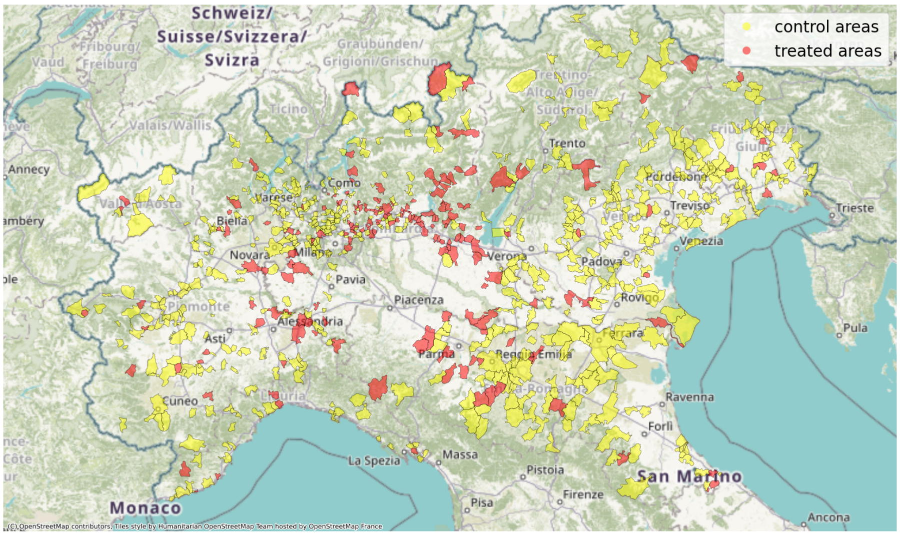

Figure S4: Placebo test cities under lockdown (the treated group - in *red*) and controls (in *yellow*).

define a binary variable *sim* indicating whether the perception of risk in the municipality is close to that of the first red zone: the positive label 1 indicates all those municipalities with Euclidean distance less or equal to the 10th percentile of the distribution of Euclidean distance values in the tweets sample (i.e. less or equal to 92.15708), the negative label 0 stands for all municipalities with distance equal or above the 90th percentile (i.e. above 113.2909).

The observations in the middle are removed to obtain a clean effect and not end up comparing two groups with gradually homogeneous increase in total deaths (Figure S4). The municipalities with positive (negative) similarity are 344 (404) and account for 6,866 (7,049) observations.

We compare the national lockdown effect on public interests displayed on Twitter between these set of municipalities with $sim = 1$ and the municipalities featuring $sim = 0$ coming from the same regions. We include a time dummy *Post* that equals 1 starting from March 9, following the government's decision to implement nationwide partial restrictions. To control for unobserved time-invariant heterogeneity arising from the large number of different and far-apart municipalities included in the sample, we include municipality level fixed-effects. From Table S7, we have no significant effect of the treatment, suggesting that indeed the significant estimates we found in the baseline models are robust anticipatory effects.

	Uncertainty					Negative Sentiment				
	(1) Aggregate	(2) Economics	(3) Health	(4) Politics	(5) Policy	(6) Aggregate	(7) Economics	(8) Health	(9) Politics	(10) Policy
post=1	-0.0613	0.00337	-0.0546	-0.00798	-0.0120	-0.0450	0.000880	-0.0438	-0.00537	0.00994
	(0.0158)	(0.00567)	(0.0154)	(0.00459)	(0.00544)	(0.0130)	(0.00427)	(0.0103)	(0.00437)	(0.00344)
sim=1 × post=1	0.00596	-0.00342	0.00883	0.0108	-0.00743	-0.00808	-0.00218	0.000716	-0.00420	-0.00752
	(0.0210)	(0.00773)	(0.0222)	(0.00582)	(0.00949)	(0.0213)	(0.00564)	(0.0195)	(0.00711)	(0.00510)
Constant	0.0369	0.0000380	0.0305	-0.00186	0.0130	0.258	0.112	0.251	0.117	-0.00162
	(0.00926)	(0.00350)	(0.0107)	(0.00238)	(0.00518)	(0.0113)	(0.00245)	(0.0110)	(0.00374)	(0.00251)
Observations	13915	13915	13915	13915	13915	13915	13915	13915	13915	13915
Adjusted R^2	0.048	0.007	0.062	-0.007	0.004	0.092	0.028	0.067	0.020	0.019
Clustered SE	Yes	Yes	Yes	Yes	Yes	Yes	Yes	Yes	Yes	Yes

Standard errors in parentheses

Table S7: Placebo Test regression table for *Uncertainty* and *Negative Sentiment*, aggregated and grouped by topics, with municipality level fixed effects (omitted). Standard error are clustered at the municipality level (in parenthesis).

G DiD Model on *Other-related* Topics

The significant negative effect of the treatment on the red zone's negative sentiment is explained by a change in emotions in discussions not related to our main topics of interest, which is a category that we can broadly describe as *leisure*, mostly pertaining to tweets on sports, music and entertainment. Diff-in-Diff coefficient estimates are given in Table S8.

	Uncertainty	Negative Sentiment
	(1) Leisure	(2) Leisure
post=1	-0.0122	-0.0562
	(0.00719)	(0.0181)
post=2	-0.0183	-0.0741
	(0.0109)	(0.0185)
redzone=1 × post=1	0.0777	-0.130
	(0.0349)	(0.0249)
redzone=1 × post=2	0.0389	-0.0937
	(0.0395)	(0.0436)
Constant	0.979	0.168
	(0.0380)	(0.0394)
Observations	24261	24261
Adjusted R^2	0.019	0.066

Standard errors in parentheses

Table S8: DiD Regression table for *Uncertainty* and *Negative Sentiment* by *other-related* topics, or Leisure, with user fixed effects. Standard error are clustered at the municipality level (in parenthesis).

	Uncertainty					Negative Sentiment				
	(1) Aggregate	(2) Economics	(3) Health	(4) Politics	(5) Policy	(6) Aggregate	(7) Economics	(8) Health	(9) Politics	(10) Policy
Bergamo	0.156	0.00525	0.0364	0.0109	0.0270	-0.0717	-0.0112	0.0266	0.0249	0.0249
	(0.0322)	(0.00743)	(0.00652)	(0.00567)	(0.00594)	(0.0272)	(0.00888)	(0.0212)	(0.0110)	(0.0201)
Brescia	0.153	0.00478	0.0376	0.0116	0.0274	-0.0925	-0.0133	0.0255	0.0205	0.0246
	(0.0326)	(0.00755)	(0.00669)	(0.00627)	(0.00599)	(0.0167)	(0.00936)	(0.0214)	(0.00954)	(0.0202)
Cremona	0.153	0.00809	0.0384	0.0104	0.0250	-0.0699	-0.00479	0.0297	0.0245	0.0259
	(0.0319)	(0.00719)	(0.00691)	(0.00458)	(0.00583)	(0.0353)	(0.00791)	(0.0215)	(0.0115)	(0.0202)
Lodi	0.150	0.00373	0.0308	0.0138	0.0266	-0.0633	-0.0129	0.0211	0.0305	0.0229
	(0.0327)	(0.00771)	(0.00533)	(0.00708)	(0.00622)	(0.0327)	(0.0102)	(0.0209)	(0.0128)	(0.0202)
Milano	0.155	0.00537	0.0360	0.0121	0.0268	-0.0722	-0.0116	0.0268	0.0259	0.0249
	(0.0321)	(0.00742)	(0.00649)	(0.00587)	(0.00594)	(0.0270)	(0.00889)	(0.0211)	(0.0110)	(0.0201)
Parma	0.155	0.00521	0.0360	0.0120	0.0265	-0.0720	-0.0117	0.0268	0.0256	0.0247
	(0.0321)	(0.00743)	(0.00649)	(0.00586)	(0.00594)	(0.0269)	(0.00889)	(0.0211)	(0.0110)	(0.0202)
Pavia	0.155	0.00540	0.0353	0.0125	0.0265	-0.0707	-0.0117	0.0283	0.0265	0.0247
	(0.0321)	(0.00740)	(0.00655)	(0.00597)	(0.00593)	(0.0272)	(0.00890)	(0.0211)	(0.0112)	(0.0201)
Piacenza	0.161	0.00327	0.0375	0.0133	0.0258	-0.0620	-0.0168	0.0298	0.0278	0.0242
	(0.0329)	(0.00750)	(0.00790)	(0.00753)	(0.00621)	(0.0341)	(0.00876)	(0.0217)	(0.0138)	(0.0202)
Clustered SE	Yes	Yes	Yes	Yes	Yes	Yes	Yes	Yes	Yes	Yes

Standard errors in parentheses

Table S9: DiD Regression estimates of $redzone = 1 \times post = 1$ for *Uncertainty* and *Negative Sentiment*, aggregated and grouped by topics with user fixed effects, dropping tweets from one administrative unit at the time. Standard error are clustered at the municipality level (in parenthesis).

H Exclusion of Territorial Administrative Units

To check whether our estimates of the effect of the lockdown are robust against the exclusion of municipalities belonging to the territorial administrative units (*province* in Italian) in the control group, we re-estimate equation the DiD model equation – equation (1) in the main text – by iterating through administrative units and dropping tweets from one control administrate unit at a time. From Table S9, the significance of the effect on uncertainty towards politics and aggregated negative sentiment depends crucially on the administrative unit included in the control group.

I Model estimates with Heteroskedasticity Robust Standard Errors

We report the DID model estimates with White standard errors robust to heteroskedasticity [9]. From Table S10, once the estimated standard errors do not account for within-city correlations, we can no longer find a significant treatment effect on political uncertainty and aggregated negative sentiment. The variance *within* clusters is much different from

the variance *between* clusters, even as we correct for violation of homoskedastic error variance.

	Uncertainty					Negative Sentiment				
	(1) Aggregate	(2) Economics	(3) Health	(4) Politics	(5) Policy	(6) Aggregate	(7) Economics	(8) Health	(9) Politics	(10) Policy
post=1	0.0199	-0.000563	0.0369	-0.00774	0.0107	-0.0443	-0.00425	0.0363	-0.0155	0.00581
	(0.00888)	(0.00251)	(0.00352)	(0.00267)	(0.00201)	(0.0102)	(0.00309)	(0.00357)	(0.00414)	(0.00186)
post=2	0.0218	0.00458	0.0419	-0.00632	0.0101	-0.0562	0.00369	0.0379	-0.0202	0.00666
	(0.00959)	(0.00283)	(0.00413)	(0.00278)	(0.00212)	(0.0109)	(0.00353)	(0.00421)	(0.00437)	(0.00200)
redzone=1 × post=1	0.155	0.00521	0.0360	0.0120	0.0265	-0.0720	-0.0117	0.0268	0.0256	0.0247
	(0.0369)	(0.00944)	(0.0169)	(0.00757)	(0.0122)	(0.0423)	(0.0142)	(0.0193)	(0.00973)	(0.0125)
redzone=1 × post=2	0.0302	-0.00378	-0.0231	0.00138	0.00529	-0.0495	-0.00606	0.00484	0.0285	0.0181
	(0.0397)	(0.0148)	(0.0151)	(0.00790)	(0.0119)	(0.0454)	(0.0169)	(0.0174)	(0.00967)	(0.0121)
Constant	0.948	-0.000804	-0.0188	0.00494	-0.0154	0.106	0.00237	-0.0427	-0.00831	-0.0247
	(0.0386)	(0.0145)	(0.0145)	(0.00739)	(0.0117)	(0.0441)	(0.0165)	(0.0169)	(0.00863)	(0.0119)
Observations	24261	24261	24261	24261	24261	24261	24261	24261	24261	24261
Adjusted R^2	0.047	0.038	0.130	0.025	0.042	0.098	0.035	0.107	0.045	0.058

Standard errors in parentheses

Table S10: DiD Regression table for *Uncertainty* and *Negative Sentiment*, aggregated and grouped by topics with user fixed effects (omitted) with White Standard Errors (in parenthesis).

J DiD Estimates Varying for Spatial Proximity

In this next section, we estimate the effect of the lockdown on the public's emotions at varying distances from the red zone, allowing us to distinguish between the effects of physical proximity and media exposure. To address the relevant changes in the Italian governmental response to the COVID-19 outbreak of early 2020, we distinguish between (i) the extended orange zone, as described by the governmental decree of March 8, 2020, and (ii) and other Northern Italian locations, which were only later subjected to nationwide measures (starting from March 9, 2020), without ever having been a targeted priority, which we refer to as *white zone*. In other words, we are interested in the following estimand:

$$\mathbb{E}[Y_{t=1}(RedZone) - Y_{t=0}(RedZone)] - \mathbb{E}[Y_{t=1}(WhiteZone) - Y_{t=0}(WhiteZone)]$$

for the difference with white zone cities, and

$$\mathbb{E}[Y_{t=1}(RedZone) - Y_{t=0}(RedZone)] - \mathbb{E}[Y_{t=1}(OrangeZone) - Y_{t=0}(OrangeZone)]$$

for the comparison with the extended orange zone. As shown in Figure S5, the extended orange zone encompassed all municipalities in Lombardia, as well as some cities that are slightly further apart from the red zone, in Emilia-Romagna and Veneto. The *white zone* is much more expanded and reaches far out in the Italian territory, connecting France to Slovenia.

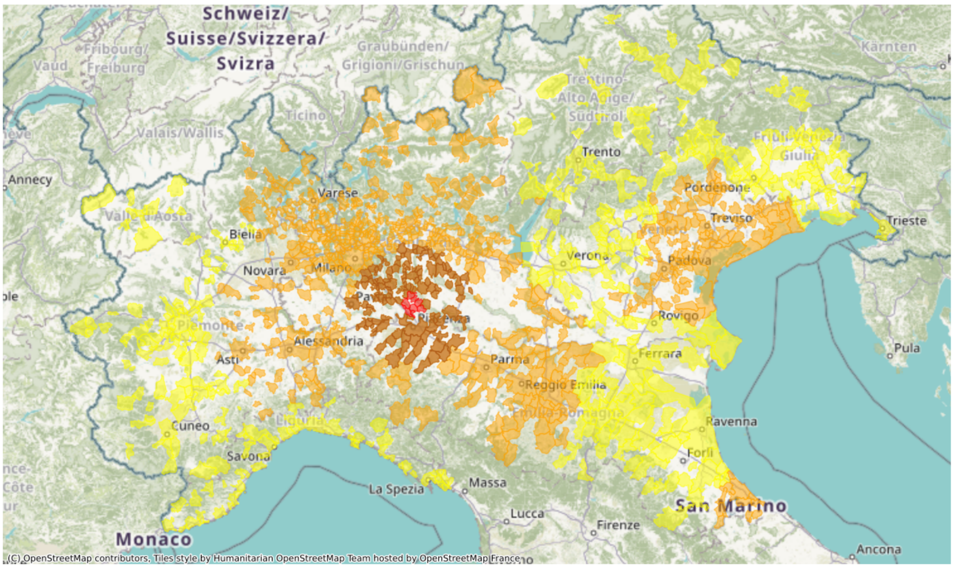

Figure S5: Sampled locations from red zone (red), orange zone units within 42km – the original controls (brown), the extended orange zone (orange), and other locations (yellow) from Northern Italy.

We compare the changes in emotions in tweets before and after the policy of February 23, 2020, from the red zone and set of controls with varying spatial proximity and exposure to governmental policy. We focus on Northern Italy to enhance socio-economic and cultural homogeneity between the comparison groups. The red zone and any city in Northern Italy are still equally influenced by the same scarce media coverage on the first lockdown, which cancels out as we remove all common confounding trends from our effect of interest in the DiD model. Unfortunately, our sample does not allow for an analysis of the treatment effect when comparing reactions in the red zone and outside of Italian boundaries, as our data does not feature tweets from locations other than in Italy. Nevertheless, it would be an interesting extension of the model. Moreover, in our data collection on Twitter, geo-filtering of GPS coordinates was applied to recover all tweets in the immediate surroundings of Codogno – within 42km. To ensure we identified tweets from within this area by matching the manually-entered user location of tweets to Istat geo-referenced data. Therefore, as we move to analyze the differences in reactions

between the red zone and controls that are increasingly distant from the red zone, our precision in the correct definition of a location suffers from a lack of filtering through GPS coordinates, leaving the user manually-entered location as the only source of information to allocate the tweets. Nevertheless, we are still able to clearly separate between tweets coming from within and outside of the red zone.

We retrieved 28 211 tweets from 465 municipalities and 5 144 unique users in the white zone and 77 979 tweets from 773 locations, and extended orange zone cities from 9 718 unique users. To prioritize computational scalability in model estimation with user fixed effects, we randomly selected 20% of observations from the extended orange zone, including 401 municipalities and 4070 unique users, and 50% of tweets from the white zone, corresponding to 295 municipalities and 3 182 users. Checking for pre-treatment covariate balance of population and economic indices at the municipality, complete randomization of the treatment status does not hold for all covariates as we consider either the full sample of observations from the extended orange zone (Figures S6), from the white zone (Figure S7), and the derived sub-samples (Figure S8 and Figure S9). With respect to the analytical sample of the main analysis, as depicted in Figure 2 of the main text, the red zone no longer has comparable pre-treatment demographic and socio-economic characteristics with the selected controls, which discourages assuming that the treatment policy is randomly allocated between the two groups. Therefore, the initial sample of controls that we carefully selected for the main analysis is small but guarantees high internal validity of our results. Increasing the sample and expanding the geographical scope for the identification of controls may lead to more generalizable results but at the cost of possibly losing causal identification of the effect of interest.

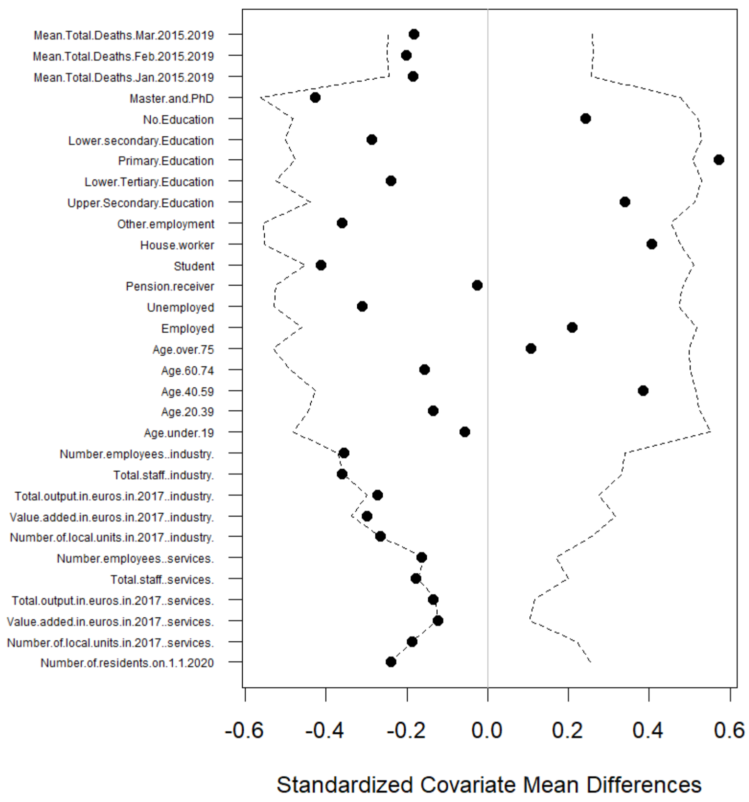

Figure S6: Standardized covariate mean difference along with 7.5% and 92.5% complete randomization quantiles using 2000 permutations. We check for covariate balance among the red zone and the extended orange zone matching the user location of tweets (8 red zone and 773 orange zone municipalities). We considered Istat data on social, economic, and demographic characteristics.

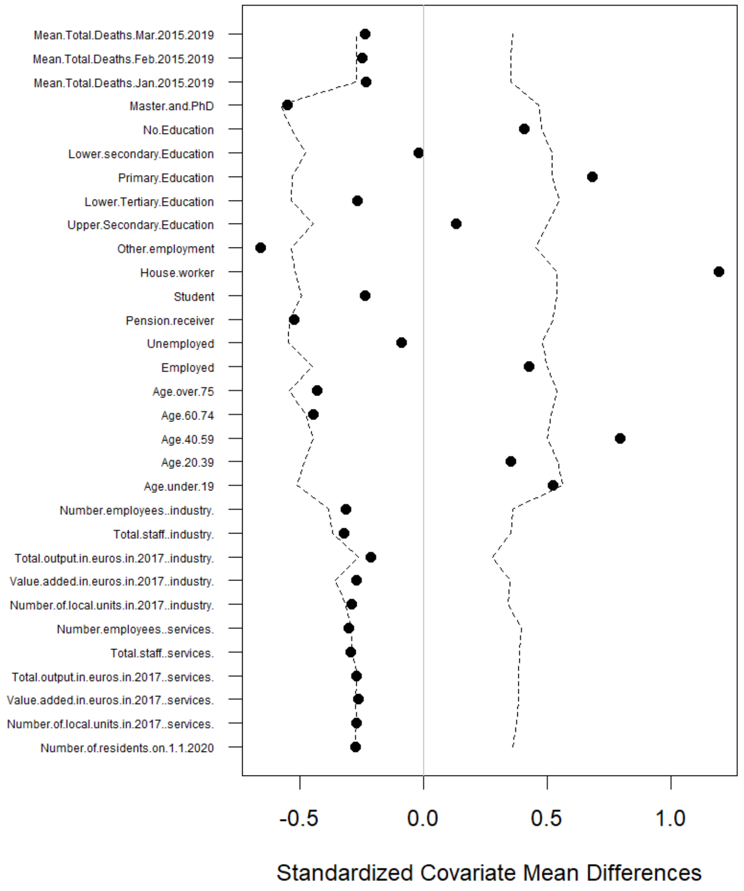

Figure S7: Standardized covariate mean difference along with 7.5% and 92.5% complete randomization quantiles using 2000 permutations. We check for covariate balance among the red zone and white zone municipalities matching the user location of tweets (8 red zone and 465 white zone municipalities). We considered Istat data on social, economic, and demographic characteristics.

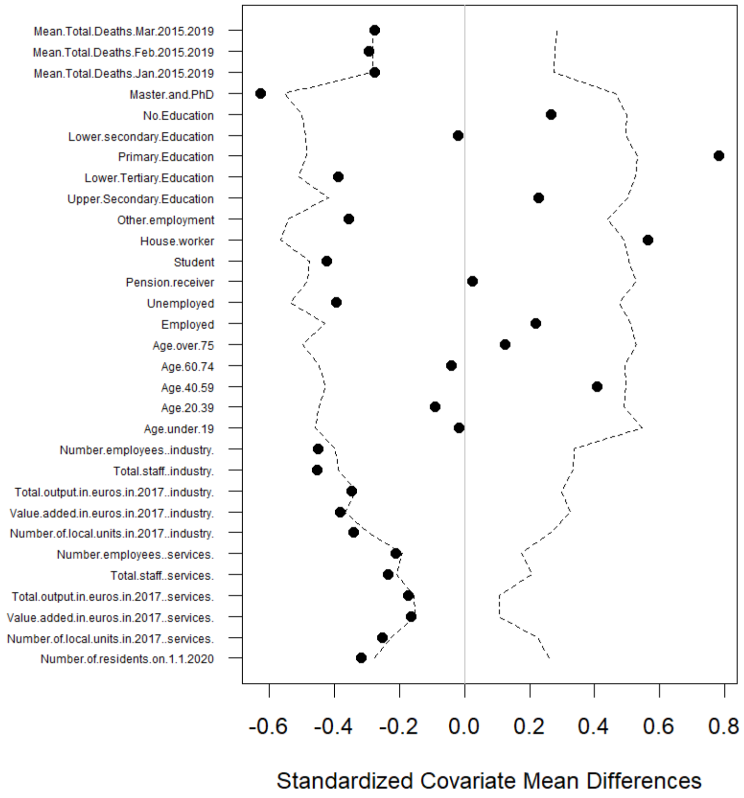

Figure S8: Standardized covariate mean difference along with 7.5% and 92.5% complete randomization quantiles using 2000 permutations. We check for covariate balance among the red zone and randomly drawn observations from the extended orange zone (20%), matching the user location of tweets (8 red zone and 401 orange zone municipalities). We considered Istat data on social, economic, and demographic characteristics.

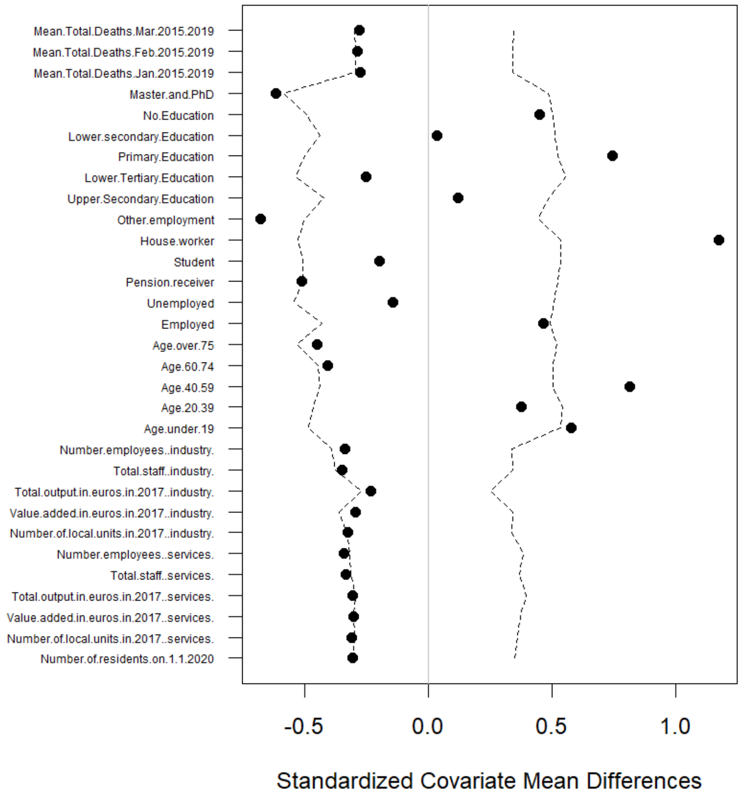

Figure S9: Standardized covariate mean difference along with 7.5% and 92.5% complete randomization quantiles using 2000 permutations. We check for covariate balance among the red zone and randomly drawn observations from the white zone (50%), matching the user location of tweets (8 red zone and 295 white zone municipalities). We considered Istat data on social, economic, and demographic characteristics.

Tables S11 and S12 report the regression model estimates when under-sampling control units from the extended orange zone and the white zone, respectively. Given the smaller sample size, we include user-level fixed effects to control for unobserved heterogeneity among users. In Table S11, the red zone expresses significantly higher uncertainty in general (0.139), and in topical discussion on health (0.031), while negative sentiments significantly decrease as a whole (-0.061), while they increase in relations to politics (0.0276). These results are in line with the findings of the baseline model in Table 2 and Table 3 of the main text. With respect to the baseline estimates of the effect in Table 2 and Table 3 of the main text, the impact of the lockdown on the public's reactions becomes less relevant as we move further away from the red zone, except for negative sentiment towards politics, as the estimated treatment effect is greater when the red zone is compared to the extended orange zone. In Table S12, we see that again aggregated

uncertainty increases significantly (0.30), and the effect is much more pronounced than in the comparison with the original set of orange zone controls as shown in Table 3 of the main text. The magnitude of the estimated effect on aggregated uncertainty (0.30), negative sentiments (-0.22), and health-related uncertainty (0.24) is greater in the white zone than in the extended orange zone. Once again, the lockdown effect on negative sentiments in discussions on politics is much larger and statistically significant when compared to extended orange zone locations.

To sum up, considering the regressions with under-sampling and user fixed effects, the baseline DiD effect in Table 2 and 3 on aggregated uncertainty, health uncertainty, and aggregated negative sentiment is confirmed, with a larger magnitude in the white zone but smaller in the extended orange zone. The effect on negative sentiments in politics significant and more pronounced in the extended orange zone compared to the white zone. However, the effect on policy loses significance as we move away from the original controls located within 42km.

	Uncertainty					Negative Sentiment				
	(1) Aggregate	(2) Economics	(3) Health	(4) Politics	(5) Policy	(6) Aggregate	(7) Economics	(8) Health	(9) Politics	(10) Policy
post=1	0.0299	0.00211	0.0416**	0.00326	0.0182**	-0.0575**	-0.00179	0.0402***	-0.0172	0.00492
	(0.0236)	(0.00834)	(0.0128)	(0.00588)	(0.00558)	(0.0218)	(0.00869)	(0.00912)	(0.00961)	(0.00473)
post=2	0.0179	0.00752	0.0185	0.00359	0.0128*	-0.0734**	0.00263	0.0275**	-0.0159	0.0146**
	(0.0312)	(0.00779)	(0.0197)	(0.00637)	(0.00628)	(0.0251)	(0.00767)	(0.00848)	(0.0103)	(0.00494)
RedvsOrangeZoneExtended=1 × post=1	0.139**	0.00260	0.0313*	0.00108	0.00625	-0.0612*	-0.0145	0.0215	0.0276*	0.0254
	(0.0439)	(0.0118)	(0.0136)	(0.00738)	(0.0138)	(0.0281)	(0.0118)	(0.0251)	(0.0128)	(0.0247)
RedvsOrangeZoneExtended=1 × post=2	0.0392	-0.00595	0.00302	-0.00862	0.0118	-0.0263	-0.00486	0.0178	0.0245	0.0107
	(0.0602)	(0.0131)	(0.0211)	(0.00888)	(0.0106)	(0.0595)	(0.0148)	(0.0257)	(0.0133)	(0.0152)
Constant	-0.0179	-0.00752	-0.0185	-0.00359	-0.0128*	0.0734**	-0.00263	-0.0275**	0.0159	-0.0146**
	(0.0312)	(0.00779)	(0.0197)	(0.00637)	(0.00628)	(0.0251)	(0.00767)	(0.00848)	(0.0103)	(0.00494)
Observations	14828	14828	14828	14828	14828	14828	14828	14828	14828	14828
Adjusted R^2	0.098	0.117	0.269	0.017	0.089	0.166	0.140	0.304	0.081	0.159

Standard errors in parentheses
* $p < 0.05$, ** $p < 0.01$, *** $p < 0.001$

Table S11: DiD Regression estimates with *RedvsOrangeZoneExtended* as treatment indicator with under-sampling of observations of extended orange zone (20%); we include user fixed effects (omitted) cluster Standard Errors by location (in parenthesis).

	Uncertainty					Negative Sentiment				
	(1) Aggregate	(2) Economics	(3) Health	(4) Politics	(5) Policy	(6) Aggregate	(7) Economics	(8) Health	(9) Politics	(10) Policy
post=1	-0.139	0.00712	-0.167	0.0210	0.0439	0.101	-0.00901	0.0388	0.0204	0.0126
	(0.104)	(0.0221)	(0.104)	(0.0122)	(0.0271)	(0.0610)	(0.0344)	(0.0631)	(0.0271)	(0.0101)
post=2	-0.163	0.0124	-0.196	0.0183	0.0285	0.0666	-0.00967	0.0119	0.00143	0.0174
	(0.107)	(0.0243)	(0.103)	(0.0141)	(0.0273)	(0.0638)	(0.0341)	(0.0662)	(0.0378)	(0.0105)
RedvsWhiteZone=1 × post=1	0.309**	-0.00241	0.240*	-0.0167	-0.0195	-0.220***	-0.00730	0.0229	-0.00993	0.0177
	(0.110)	(0.0236)	(0.104)	(0.0130)	(0.0298)	(0.0634)	(0.0353)	(0.0672)	(0.0284)	(0.0259)
RedvsWhiteZone=1 × post=2	0.220	-0.0108	0.217*	-0.0233	-0.00391	-0.166*	0.00744	0.0334	0.00716	0.00803
	(0.118)	(0.0264)	(0.103)	(0.0154)	(0.0285)	(0.0830)	(0.0363)	(0.0704)	(0.0387)	(0.0176)
Constant	0.163	-0.0124	0.196	-0.0183	-0.0285	-0.0666	0.00967	-0.0119	-0.00143	-0.0174
	(0.107)	(0.0243)	(0.103)	(0.0141)	(0.0273)	(0.0638)	(0.0341)	(0.0662)	(0.0378)	(0.0105)
Observations	14207	14207	14207	14207	14207	14207	14207	14207	14207	14207

Standard errors in parentheses
* $p < 0.05$, ** $p < 0.01$, *** $p < 0.001$

Table S12: DiD Regression estimates with *RedvsWhiteZone* as treatment indicator with under-sampling of observations of white zone (50%); we include user fixed effects (omitted) cluster Standard Errors by location (in parenthesis).

To evaluate how the lockdown effect changes as we vary the spatial distance to the red zone, we modify the model in equation (1) of the main text to replace the treatment binary *red zone* indicator with a new categorical variable: *RingOrange* identifies rings of extended orange zone locations with increasing distance from the red zone (centered on the red zone), while with *RingWhite* we identify rings of white zone locations of increasing distance, centered on the red zone. $RingOrange = 0$ ($RingWhite = 0$) identifies the red zone and is the baseline level of the regression model; the first ring identifies tweets from locations within 42km from Codogno, from the extended orange zone ($RingOrange = 1$), and the white zone ($RingWhite = 1$), but we have no observations from the first ring of the white zone, as it should be. The second tier is ($RingOrange = 2$ and $RingWhite = 2$) between 42km and 100km. At last, $RingOrange = 3$ and $RingWhite = 3$ identifies tweets beyond 100km from Codogno, within the extended orange zone and the white zone respectively. We are interested in the coefficient estimates of $post = 1 \times RingOrange$ for the comparing with extended orange zone locations, and $post = 1 \times RingWhite$ for the difference in public reactions between red zone and white zone, indicating the post-treatment variation in emotions in tweets in the given ring when compared to the red zone (baseline). Tables S13-S14, we report the coefficient estimates of the treatment effect as we increase the spatial distance of the comparison group, considering randomly drawn samples of tweets from the extended orange zone (Table S13) and from the white zone (Table S14).

Considering the randomly selected sub-sample of tweets from the extended orange

zone (Table S13), the orange zone displays less aggregated uncertainty (column 1) and uncertainty related to health (column 2) with respect to the red zone, and the difference increases with distance (post=1 × RingOrange). On the other hand, while the difference in aggregated negative sentiments increases with distance (column 6), the difference in negative sentiments related to politics (column 9) decreases as we move away from the red zone. Moving to the white zone locations (Table S14), the most signal comes from the second tier of locations, between 42km and 100 km from Codogno. In general, the effect (post=1 × RingWhite) loses its impact as we increase the distance from the red zone. Interestingly, we find a significant difference between red zone and white zone locations in economic negative sentiments. Moreover, while aggregated negative sentiments were higher in the extended orange zone cities of the second ring (above 42km of distance to Codogno) than in the red zone after the policy, tweets from the same second tier in the white zone instead are less likely to display negative sentiments when compared to the red zone.

For an overall discussion of the results, see the Robustness Checks Section of the main text.

	Uncertainty					Negative Sentiment				
	(1) Aggregate	(2) Economics	(3) Health	(4) Politics	(5) Policy	(6) Aggregate	(7) Economics	(8) Health	(9) Politics	(10) Policy
post=1	0.169***	0.00472	0.0729***	0.00434	0.0244	-0.119***	-0.0163*	0.0617**	0.0104	0.0303
	(0.0371)	(0.00839)	(0.00470)	(0.00445)	(0.0126)	(0.0177)	(0.00802)	(0.0234)	(0.00847)	(0.0243)
post=2	0.0570	0.00157	0.0215**	-0.00503	0.0246**	-0.0997	-0.00222	0.0453	0.00859	0.0254
	(0.0515)	(0.0106)	(0.00760)	(0.00619)	(0.00855)	(0.0540)	(0.0126)	(0.0242)	(0.00852)	(0.0144)
RingOrange=2	0.264	0.0485	0.285	0.0133	0.0421	1.081***	0.0209	0.979**	0.00136	-0.174
	(0.199)	(0.0427)	(0.198)	(0.0291)	(0.0536)	(0.300)	(0.0154)	(0.302)	(0.0169)	(0.225)
post=1 × RingOrange=1	-0.144**	-0.00304	-0.0251	-0.00354	-0.0106	0.0564*	0.0166	-0.0270	-0.0293*	-0.0229
	(0.0450)	(0.0128)	(0.0136)	(0.00813)	(0.0136)	(0.0275)	(0.0123)	(0.0246)	(0.0138)	(0.0248)
post=1 × RingOrange=2	-0.148*	0.00139	-0.116**	0.0123	0.0163	0.129*	-0.00646	0.0173	-0.0213	-0.0167
	(0.0663)	(0.00987)	(0.0390)	(0.00868)	(0.0143)	(0.0578)	(0.0158)	(0.0269)	(0.0177)	(0.0254)
post=1 × RingOrange=3	-0.367*	0.0279	-0.295	0.00996	0.0495	0.201	0.0177	-0.0311	-0.00344	-0.203
	(0.175)	(0.0444)	(0.174)	(0.0174)	(0.0493)	(0.286)	(0.00974)	(0.303)	(0.00987)	(0.225)
post=2 × RingOrange=1	-0.0175	0.00472	0.0268*	0.00879	-0.0128	0.00484	0.00841	-0.0198	-0.0248	-0.0131
	(0.0655)	(0.0131)	(0.0129)	(0.00975)	(0.0124)	(0.0596)	(0.0153)	(0.0256)	(0.0149)	(0.0152)
post=2 × RingOrange=2	-0.0682	0.00668	-0.104*	0.0160	0.00415	0.103	-0.0154	0.00922	-0.0153	0.00123
	(0.0763)	(0.0115)	(0.0431)	(0.0105)	(0.0113)	(0.0756)	(0.0163)	(0.0283)	(0.0188)	(0.0163)
post=2 × RingOrange=3	-0.271	0.0530	-0.279	0.0350	0.0582	0.191	0.000363	0.0125	-0.0181	-0.186
	(0.199)	(0.0437)	(0.194)	(0.0288)	(0.0538)	(0.300)	(0.0146)	(0.302)	(0.0108)	(0.225)
Constant	-0.253	-0.0567	-0.203	-0.0243	-0.0709	-1.084***	-0.00327	-1.033***	0.00531	0.147
	(0.192)	(0.0432)	(0.194)	(0.0284)	(0.0533)	(0.295)	(0.00891)	(0.301)	(0.00763)	(0.225)
Observations	14828	14828	14828	14828	14828	14828	14828	14828	14828	14828

Standard errors in parentheses

* $p < 0.05$, ** $p < 0.01$, *** $p < 0.001$

Table S13: Regression model estimates on $post = 1 \times RingOrange$ for red zone and extended orange zone observations, under-sampling of observations of orange zone (20%); we include user fixed effects (omitted) cluster Standard Errors by location (in parenthesis).

	Uncertainty					Negative Sentiment				
	(1)<br>Aggregate	(2)<br>Economics	(3)<br>Health	(4)<br>Politics	(5)<br>Policy	(6)<br>Aggregate	(7)<br>Economics	(8)<br>Health	(9)<br>Politics	(10)<br>Policy
post=1	0.169***	0.00472	0.0729***	0.00434	0.0244*	-0.119***	-0.0163*	0.0617**	0.0104	0.0303
	(0.0364)	(0.00824)	(0.00461)	(0.00438)	(0.0124)	(0.0174)	(0.00788)	(0.0230)	(0.00832)	(0.0238)
post=2	0.0570	0.00157	0.0215**	-0.00503	0.0246**	-0.0997	-0.00222	0.0453	0.00859	0.0254
	(0.0506)	(0.0104)	(0.00747)	(0.00608)	(0.00840)	(0.0531)	(0.0124)	(0.0238)	(0.00837)	(0.0141)
post=1 × RingWhite=2	-0.769***	-0.00472	-0.673***	-0.00434	-0.0244*	-0.481***	0.0163*	-0.662***	-0.0104	-0.0303
	(0.0364)	(0.00824)	(0.00461)	(0.00438)	(0.0124)	(0.0174)	(0.00788)	(0.0230)	(0.00832)	(0.0238)
post=1 × RingWhite=3	-0.301**	0.00237	-0.233*	0.0161	0.0203	0.234***	0.00738	-0.0106	0.0108	-0.0173
	(0.111)	(0.0240)	(0.105)	(0.0132)	(0.0302)	(0.0635)	(0.0359)	(0.0669)	(0.0287)	(0.0260)
post=2 × RingWhite=2	-0.836***	-0.0273	-0.756***	-0.185***	-0.0365**	-0.316**	0.0478**	-0.562***	0.0579	0.00759
	(0.0833)	(0.0208)	(0.0509)	(0.0549)	(0.0128)	(0.101)	(0.0178)	(0.0798)	(0.0406)	(0.0171)
post=2 × RingWhite=3	-0.211	0.0111	-0.209*	0.0247	0.00469	0.178*	-0.00786	-0.0224	-0.00726	-0.00796
	(0.119)	(0.0267)	(0.105)	(0.0156)	(0.0289)	(0.0829)	(0.0369)	(0.0699)	(0.0392)	(0.0177)
Constant	0.154	-0.0127	0.187	-0.0197	-0.0293	-0.0781	0.0101	-0.0229	-0.00132	-0.0174
	(0.108)	(0.0247)	(0.104)	(0.0144)	(0.0277)	(0.0637)	(0.0347)	(0.0657)	(0.0383)	(0.0107)
Observations	14207	14207	14207	14207	14207	14207	14207	14207	14207	14207

Standard errors in parentheses
* $p < 0.05$, ** $p < 0.01$, *** $p < 0.001$

Table S14: Regression model estimates on $post = 1 \times RingWhite$ for red zone and white zone observations, under-sampling of observations of white zone (50%); we include user fixed effects (omitted) cluster Standard Errors by location (in parenthesis).

References

[1] J. S. Pischke. "Empirical Methods in Applied Economics". In: *LSE Lecture Notes* (2005). URL: https://econ.lse.ac.uk/staff/spischke/ec524/evaluation3.pdf.

[2] Ariella Kahn-Lang and Kevin Lang. "The Promise and Pitfalls of Differences-in-Differences: Reflections on 16 and Pregnant and Other Applications". In: *Journal of Business & Economic Statistics* 38.3 (2020), pp. 613–620. DOI: 10.1080/07350015.2018.1546591.

[3] Donald B. Rubin. "Comment". In: *Journal of the American Statistical Association* 81.396 (1986), pp. 961–962. DOI: 10.1080/01621459.1986.10478355.

[4] Paul R Rosenbaum. "Interference Between Units in Randomized Experiments". In: *Journal of the American Statistical Association* 102.477 (2007), pp. 191–200. DOI: 10.1198/016214506000001112.

[5] Kyle Butts. *Difference-in-Differences Estimation with Spatial Spillovers*. Papers. arXiv.org, 2023. URL: https://EconPapers.repec.org/RePEc:arx:papers:2105.03737.

[6] Yoav Benjamini and Yosef Hochberg. "Controlling The False Discovery Rate - A Practical And Powerful Approach To Multiple Testing". In: *J. Royal Statist. Soc., Series B* 57 (1995), pp. 289–300. DOI: 10.2307/2346101.

[7] ISTAT. "Tavola decessi per 6.866 comuni al 31marzo2020". In: *Istituto nazionale di statistica e informatica* (2020), (accessed on July 9, 2022). URL: https://www.istat.it/it/archivio/240401.

[8] ISTAT. "Confini delle unità amministrative a fini statistici al 1° gennaio 2022". In: *Istituto nazionale di statistica e informatica* (2022), (accessed on July 6, 2022). URL: https://www.istat.it/it/archivio/222527.

[9] Halbert White. "A heteroskedasticity-consistent covariance matrix estimator and a direct test for heteroskedasticity". In: *Econometrica: journal of the Econometric Society* (1980), pp. 817–838.
