# Supplementary figures and images for: The Impact of Stay-At-Home Mandates on Uncertainty and Sentiments: Quasi-Experimental Study
Source: J Med Internet Res. 2025 Mar 4;27:e64667. doi: 10.2196/64667 (PMC11920662; doi:10.2196/64667)

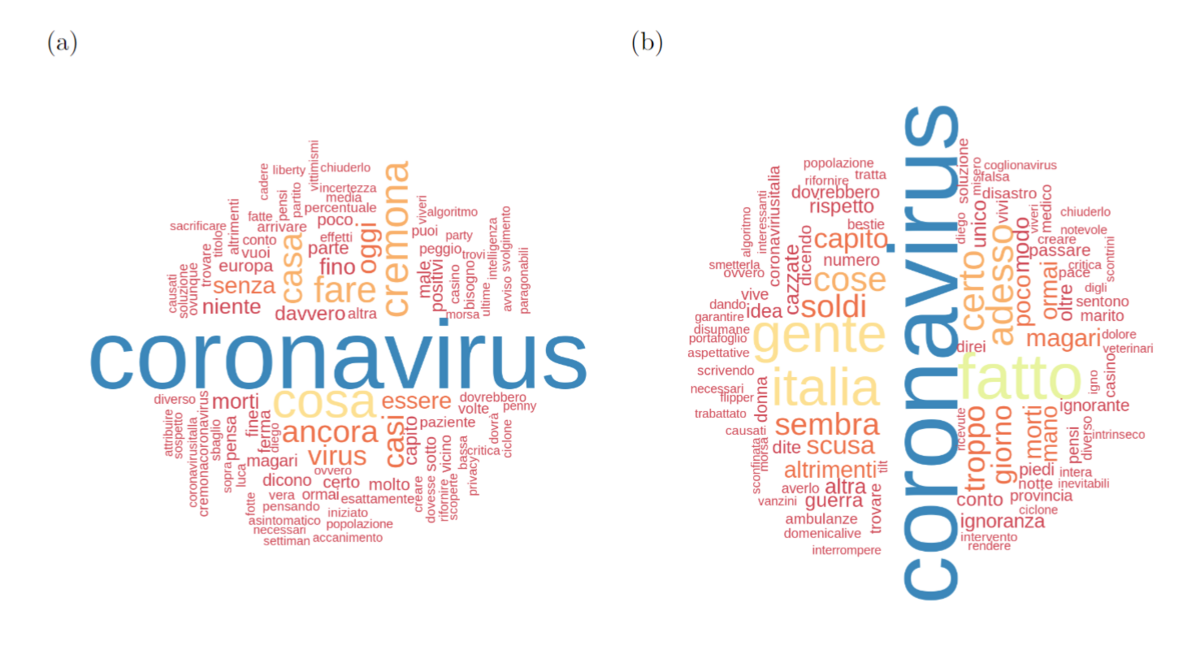

Supplement: Multimedia Appendix 2 [file jmir_v27i1e64667_app2.png]
